# Recent Advances of g-C_3_N_4_/LDHs Composite Photocatalysts in Water Pollution Treatment

**DOI:** 10.3390/molecules31010180

**Published:** 2026-01-03

**Authors:** Jing Li, Yaping Guo, Jie Bai

**Affiliations:** 1 School of Basic Medicine, North Henan Medical University, Xinxiang 453003, China; 2 College of Materials, Xiamen University, Xiamen 361005, China

**Keywords:** graphitic carbon nitride, layered double hydroxides, composite, photocatalyst, water pollution treatment

## Abstract

Water pollution poses a pressing global environmental threat, driving an urgent need for efficient, stable, and eco-friendly water treatment techniques. Semiconductor photocatalysis has emerged as a highly promising solution, utilizing solar energy to thoroughly degrade pollutants under mild conditions without secondary pollution. Among numerous photocatalysts, the graphitic carbon nitride (g-C_3_N_4_)/layered double hydroxides (LDHs) heterostructures represent a kind of high-performance photocatalysts that combine the integrated advantages of both components. These composites exhibit enhanced visible-light absorption, a highly efficient charge separation and transfer, and a significantly increased specific surface area that promotes the enrichment and degradation of pollutants. The synergistic interaction between g-C_3_N_4_ and LDHs not only mitigates their individual limitations but also creates a superior photocatalytic system with improved adsorption capacity and reaction kinetics. This review systematically summarizes recent advances in g-C_3_N_4_/LDHs composite photocatalysts for aquatic pollutant removal. It elaborates on the structural synergies, synthesis routes, and optimization strategies, with a particular focus on applications and mechanistic insights into the degradation of various pollutants-including organic dyes, drugs, and phenolics. Finally, the review outlines current challenges and future research directions, such as deepening mechanistic understanding, designing multifunctional systems, and advancing toward scalable implementation, providing a valuable reference for developing next-generation photocatalytic water treatment technologies.

## 1. Introduction

Water is indispensable to life on Earth and crucial for ecosystem integrity and sustainable human development. However, rapid industrialization and urbanization have led to severe water pollution, with emerging pollutants—including antibiotics, industrial dyes, and phenolic compounds—posing significant threats to ecosystems and human health due to their persistence, bioaccumulation, and toxicity [1,2,3,4]. Advanced oxidation processes (AOPs) have emerged as efficient strategies for eliminating such pollutants, among which heterogeneous photocatalysis stands out for its environmental benignity, reliance on solar energy, and ability to mineralize pollutants into non-toxic products (e.g., CO_2_, H_2_O) [5].

Graphitic carbon nitride (g-C_3_N_4_), a prominent metal-free semiconductor, has become a focal point in heterogeneous photocatalysis due to its visible-light-active band gap (~2.7 eV), excellent chemical stability, cost-effective precursors, and non-toxic nature [6,7,8]. Nevertheless, its practical performance is often hampered by limited visible-light absorption, a low density of active sites, and fast recombination of photogenerated charge carriers [9]. Similarly, layered double hydroxides (LDHs), a class of anionic clay materials characterized by tunable interlayer anions, high specific surface area, and abundant surface hydroxyl groups-have demonstrated remarkable potential as adsorbents and catalysts in photocatalytic systems [10,11,12]. However, the widespread application of pure LDHs is constrained by their typically wide bandgaps (restricting activity to ultraviolet light) and poor electrical conductivity [13]. The integration of g-C_3_N_4_ with LDHs to form heterostructured composites creates a synergistic “1 + 1 > 2” effect [14]. In such architectures, g-C_3_N_4_ serves as a visible-light-harvesting component, while LDHs act as co-catalysts, structural supports, or pollutant adsorbents, collectively facilitating enhanced reaction kinetics [15]. This strategic combination not only mitigates the individual limitations of each component but also establishes efficient interfacial charge-transfer pathways, thereby significantly boosting the overall photocatalytic performance [16,17,18].

In this review, we comprehensively summarizes recent advances in g-C_3_N_4_/LDHs composite photocatalysts for water treatment. We begin by outlining their structural traits and synergistic merits, followed by a discussion of synthesis methods and performance optimization strategies. We then systematically catalog their applications in removing various water pollutants and delve into the underlying photocatalytic mechanisms. Finally, we conclude by addressing current challenges and outlining promising avenues for future research. This overview aims to provide a clear framework and valuable insights for researchers, fostering further development and practical implementation of g-C_3_N_4_/LDHs-based photocatalysts.

## 2. Material Characteristics and Synergistic Advantages of g-C_3_N_4_ and LDHs

### 2.1. Structure and Photocatalytic Performance of g-C_3_N_4_

**Figure 1:**
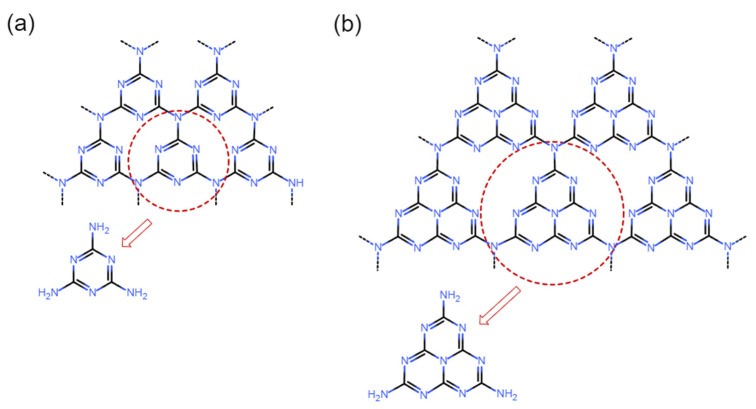
Structural configurations of the triazine moiety (**a**) and tri-s-triazine unit (**b**) within the g-C_3_N_4_ framework. In both panels (**a**,**b**), the dashed red circle labels a distinct triazine/heptazine ring core structural region of the g-C_3_N_4_, and the arrow corresponds to the bonding or assembly of the target amino-substituted aromatic unit to the labeled site.

g-C_3_N_4_ is a two-dimensional polymeric semiconductor formed by nitrogen and carbon atoms connected through sp^2^-hybridized covalent bonds [19]. Its basic structural unit consists of a honeycomb-like planar structure formed by triazine rings (s-triazine) or tri-s-triazine/heptazine rings bridged by nitrogen atoms, with these units stacked via van der Waals forces to form a graphite-like layered structure (Figure 1a,b) [20]. This unique fully conjugated π-electron system confers semiconductor properties to g-C_3_N_4_ [21]. Photocatalytic activity arises from its electronic structure—the valence band originates mainly from nitrogen’s 2p lone-pair electrons, while the conduction band comprises antibonding π* orbitals formed by *p*_z orbitals of both nitrogen and carbon atoms [22]. With an approximate band gap of 2.7 eV, this material demonstrates potential absorption capabilities for visible radiation under 460 nm [23]. Upon illumination, g-C_3_N_4_ generates electron-hole pairs. The conduction band’s redox potential (~−1.1 V vs. NHE) is negative enough to theoretically reduce O_2_ to superoxide radicals (·O_2_^−^), and the valence band potential (~+1.6 V vs. NHE) is positive enough to oxidize most organic compounds, though its ability to firsthand oxidize H_2_O to hydroxyl radicals (·OH) is limited [24].

Nevertheless, the practical photocatalytic performance of bulk g-C_3_N_4_ often falls short of theoretical predictions, mainly due to four factors: severe recombination of photoinduced carriers (electron-hole pair recombine before reaching the interface, leading to low quantum efficiency) [25]; small specific surface area (usually less than 20 m^2^/g) and insufficient catalytic sites caused by tight layer stacking [26]; limited light absorption capacity (mainly concentrating on blue-violet light, with poor utilization of longer-wavelength visible light) [27]; and low charge mobility (slow carrier migration within and between layers) [28].

### 2.2. Structure and Photocatalytic Performance of LDHs

**Figure 2:**
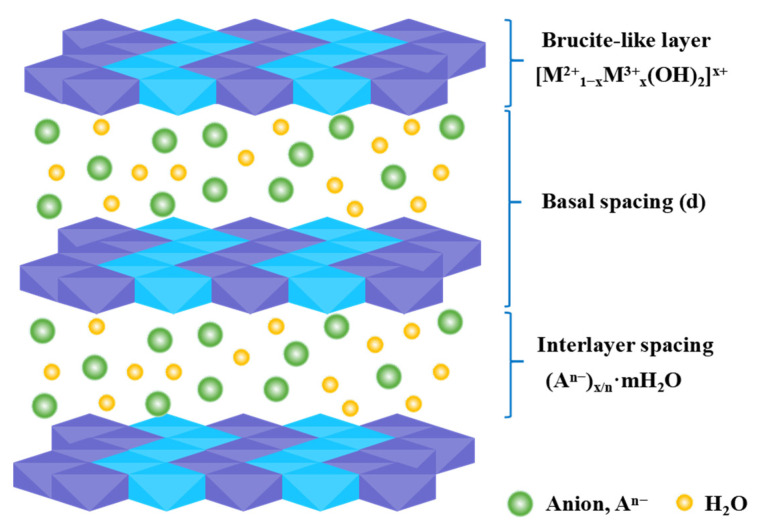
Structure of LDHs. The purple and blue polyhedral structural units in the figure correspond to the hydroxide octahedrons of metal ions of different valence states in LDHs brucite layer. The green spheres represent interlayer anions (A^2−^), and the yellow spheres represent interlayer water molecules (H_2_O).

LDHs exhibit a brucite (Mg(OH)_2_)-like structure (Figure 2), constituted of positively charged brucite-type main sheets and interlayer anions/water molecules, and follow a general chemical formula [M^2+^_1−x_M^3+^_x_(OH)_2_]^x+^·(A^n−^)_x/n_·mH_2_O [29]. The types of M^2+^ (e.g., Mg^2+^, Zn^2+^, Ni^2+^, Co^2+^) and M^3+^ (e.g., Fe^3+^, Cr^3+^, Al^3+^) in the layers, their ratio (x ≈ 0.2–0.33), and interlayer anions An^−^ (e.g., CO_3_^2−^, NO_3_^−^, Cl^−^, SO_4_^2−^) can be tailored to form materials like MgAl-LDH, ZnAl-LDH, NiFe-LDH, and CoAl-LDH [30]. This flexibility allows precise tuning of properties (e.g., layer charge density, acidity/basicity, redox capacity) [31]. Beyond common anions, functional anions (e.g., polyoxometalates, organic molecules) can be intercalated to enable multifunctionality [32].

As a photocatalytic cocatalyst, LDHs have both limitations and advantages. Most common LDHs (e.g., MgAl-LDH) show negligible intrinsic photocatalytic performance under visible/ultraviolet (UV) light due to large band gap (3.0–5.0 eV) from stable electronic configurations, resulting in limited light absorption and high carrier recombination rates [33,34]. While transition metal-containing LDHs (e.g., NiFe-LDH, NiCo-LDH) show narrower band gap of 2.0–3.4 eV, they possess inherent photocatalytic activity under appropriate light irradiation [35,36]. Band gap values of LDHs are commonly determined by ultraviolet-visible diffuse reflectance spectroscopy (UV-Vis DRS) combined with the Tauc plot method. The band gap is strongly dependent on LDHs composition: the type and molar ratio of M^2+^/M^3+^ cations regulate the electronic structure (e.g., d-d electron transitions of transition metals reduce band gap energy), while interlayer anions affect the layer charge density and interfacial electron distribution, thereby indirectly modifying the band gap [37]. Meanwhile, LDHs possess excellent adsorption and enrichment capabilities: positively charged layers and large specific surface areas enable them to adsorb anionic pollutants via electrostatic attraction and other pollutants through hydrogen bonding and van der Waals forces, which increases pollutant proximity to reactive species and enhances degradation rates [38,39]. Moreover, their alkaline sites (e.g., M-OH groups) promote ·OH generation, and they can act as electron traps or hole-transport channels to facilitate charge separation in primary photocatalysts [40,41]. Additionally, LDHs are promising precursors for mixed metal oxides (MMOs) with enhanced photocatalytic properties. Upon calcination (typically 300–800 °C), LDHs undergo thermal decomposition to form MMOs with retained high specific surface area, uniform metal distribution, and abundant oxygen vacancies, which significantly improve light absorption and charge carrier separation efficiency [42]. This characteristic is particularly relevant as calcination is later described as an effective method for preparing g-C_3_N_4_/LDHs composite photocatalysts.

### 2.3. Synergistic Advantages of Composites

The combination of g-C_3_N_4_ with LDHs to construct g-C_3_N_4_/LDHs heterojunctions fully leverages the strengths of both materials while mitigating their individual limitations, resulting in a synergistic enhancement effect where “1 + 1 > 2” [43]. In the g-C_3_N_4_/LDHs composite material, LDHs’ main functions include: (i) acting as a catalyst aid or a direct photoreactive component (depending on its band gap), participating in the generation and separation of photogenerated carriers; (ii) functioning as an efficient adsorbent, enriching target pollutants and increasing local concentration; (iii) serving as a structural support, preventing the aggregation of g-C_3_N_4_ and increasing the specific surface area. The core synergistic advantages are primarily manifested in the following aspects:

(1) Formation of heterojunctions promoting charge separation and migration. This is the most critical synergistic effect. Both LDHs and g-C_3_N_4_ are two-dimensional layered nanomaterials. Their composite is capable of forming a 2D/2D heterostructure, which expands the interfacial contact area through “face-to-face contact” and shortens the charge migration distance, thereby optimizing the spatial separation and directional movement of photoinduced charge carriers to boost catalytic performance [15]. Moreover, the band structures of LDHs and g-C_3_N_4_ are well-matched. After compositing, internal electric fields (e.g., S-Scheme) can be formed, driving counter-directional migration of electrons from g-C_3_N_4_ and holes from LDHs, effectively suppressing their recombination in the bulk [44]. Depending on the band alignment, different heterojunction types such as Type-II, Z-Scheme, or S-Scheme can be formed [45].

(2) The expanded surface area enhances the availability of active catalytic sites. Bulk g-C_3_N_4_ and layered double hydroxide (LDH) layers are prone to stacking [46,47]. Their composite formation, especially in a 2D/2D sheet structure, acts as “spacers” to prevent self-agglomeration and restacking [48]. For example, combining exfoliated g-C_3_N_4_ nanosheets with LDH nanosheets forms composites with a “house-of-cards” open structure, exhibiting a significantly larger specific surface area than individual components [49]. This exposes more catalytic active sites and facilitates pollutant adsorption and mass transfer.

(3) Synergistic adsorption-photocatalysis. The exceptional adsorption capabilities of LDHs serve as crucial functional components. LDHs first capture and concentrate low-concentration pollutants from water onto the catalyst surface. Subsequently, adjacent g-C_3_N_4_ generates highly reactive oxygen species (ROS) under light, that act on and degrade the concentrated pollutants in situ without long-distance diffusion [50]. For LDHs with intrinsic photocatalytic activity, they can simultaneously generate ROS to participate in pollutant degradation, further enhancing the synergistic effect. This “capture-then-degrade” mode significantly enhances the apparent rate and overall efficiency of photocatalytic reactions, particularly for low-concentration wastewater treatment [51]. For instance, MgAl-LDH/g-C_3_N_4_ composites demonstrate markedly improved removal efficiency for anionic dyes due to this synergy [52].

(4) Broadened spectral response range. Although g-C_3_N_4_ exhibits intrinsic visible-light responsiveness, compounding with LDHs containing specific transition metals (e.g., Fe^3+^, Co^2+^, Ni^2+^) further enhances visible-light harvesting [53]. The d-d electron transitions of transition metals in LDHs absorb specific visible-light wavelengths and transfer energy or electrons to g-C_3_N_4_ via energy or charge transfer, improving solar spectrum utilization by the composite system [54].

(5) Enhanced stability and recyclability. LDHs as carriers improve the structural stability and mechanical strength of the composite. Particularly when LDHs contain magnetic metals (e.g., Fe^3+^, Co^2+^, Ni^2+^), the composite gains magnetism, enabling easy post-reaction separation from water via an external magnetic field [55]. This resolves the challenge of recovering powdered catalysts and reduces application costs.

## 3. Preparation Methods and Performance Optimization

The morphology, interfacial contact intimacy, and synergy strength of g-C_3_N_4_/LDHs composites are closely related to preparation methods [56]. Ideal methods should ensure the formation of intimate, high-quality heterointerfaces between components to facilitate rapid charge transfer, while also being low-cost, simple, highly controllable, and easily scalable.

### 3.1. Mainstream Preparation Techniques

#### 3.1.1. Electrostatic Self-Assembly Method

This method utilizes the principle of spontaneous assembly of oppositely charged substances in solution via electrostatic attraction. Typically, g-C_3_N_4_ nanosheets obtained through thermal or liquid-phase ultrasonic exfoliation exhibit surface charges (negative at specific pH) due to protonation/deprotonation of -NH_2_ or -NH- groups, while LDH layers inherently possess permanent positive charges [57,58]. Thus, when dispersed in a solvent, they attract each other electrostatically. In a typical electrostatic self-assembly approach, if LDHs with strongly bound anions (e.g., carbonate ions) are employed, g-C_3_N_4_ nanosheets will be mainly anchored on the external surfaces of LDH nanosheets to form surface heterojunctions rather than intercalated into the interlayer spaces, since the size of g-C_3_N_4_ nanosheets is much larger than the interlayer spacing of LDH nanosheets. This surface-driven assembly mode favors the formation of tightly integrated composites. This approach is simple and mild, largely preserving the original structure and morphology of components, and facilitates the construction of closely contacted 2D/2D heterojunctions [59].

**Figure 3:**
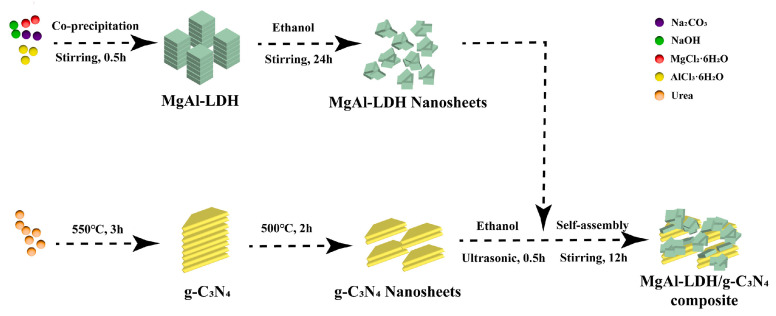
Electrostatic self-assembly process diagram of MgAl-LDH/g-C_3_N_4_. Reprinted with permission from Ref. [52].

For example, Hao et al. [52] first prepared negatively charged graphitic carbon nitride nanosheets (CNNS) via urea thermal polymerization (550 °C, 3 h) followed by thermal exfoliation (500 °C, 2 h). Positively charged MgAl-LDH nanosheets were synthesized by a modified aqueous miscible organic solvent treatment (AMOST) method using MgCl_2_·6H_2_O and AlCl_3_·6H_2_O as precursors, Na_2_CO_3_ as intercalant, and NaOH to adjust pH~9.5, yielding flower-like nanospheres composed of ultrathin nanosheets. Then utilizing electrostatic-driven assembly approach (Figure 3), MgAl-LDH/g-C_3_N_4_ composites with varying mass ratios (designated as MCN-X, X = 1, 3, 5, 7, 10) were synthesized. The formation of MgAl-LDH nanolayers was verified by X-ray diffraction (characteristic peaks of (003), (006) planes), Fourier transform infrared spectroscopy(Mg-OH vibrations at 431 cm^−1^), and transmission electron microscopy (lattice fringes of 0.17/0.19 nm corresponding to MgAl-LDH). Optimal performance was achieved at a 5:10 ratio (MCN-5), demonstrating that electrostatic self-assembly method can precisely control over component proportions. Moreover, the MgAl-LDH nanosheets formed by electrostatic self-assembly were uniformly distributed on the g-C_3_N_4_ nanosheets, increasing the pore volume and specific surface area of the composite material, thereby enhancing its adsorption performance toward methyl orange (MO). Results showed suppressed electron-hole recombination, improved photocatalytic performance, and significantly increased MO degradation efficiency (93.58% removal at optimal ratio).

Zhou et al. [60] self-assembled NiCo-LDH nanoflowers and ultrathin g-C_3_N_4_. Uniform compounding via ultrasonication and stirring formed tight heterostructures. This electrostatic self-assembly increased specific surface area and promoted interfacial charge transport. The engineered NiCo-LDH/g-C_3_N_4_ hybrid demonstrated numerous catalytic centers and Z-Scheme charge migration characteristics, significantly boosting both efficiency and product specificity in photocatalytic CO_2_ conversion. Experimental data revealed CO and CH_4_ evolution rates peaking at 114.24 and 26.48 μmol·h^−1^·g^−1^, respectively, outperforming individual components by substantial margins. These results confirmed the efficacy of the heterojunction constructed by electrostatic assembly method in facilitating charge separation.

#### 3.1.2. Co-Precipitation Method

The co-precipitation technique has become a conventional method for preparing LDHs [61]. In preparing g-C_3_N_4_/LDHs composites, pre-synthesized g-C_3_N_4_ nanomaterials are dispersed in a mixed solution of M(II) and M(III) metal salts. Metal ions are then co-precipitated at constant pH (adjusted by adding alkali solutions such as NaOH or ammonia), enabling in situ LDHs layer growth [62]. Here, g-C_3_N_4_ acts as a nucleating agent or template, with LDHs nanosheets directly growing on its surface to form “face-to-face” or “face-to-edge” close contacts [63]. This method achieves uniform, in situ LDHs growth on g-C_3_N_4_, creating tight interfaces favorable for charge transfer [64].

For instance, Sahoo et al. [65] synthesized S, P-g-C_3_N_4_/ZnCr-LDH heterocomposites via in situ co-precipitation. Specific mass percentages (5, 10, 20, 30 wt%) of g-C_3_N_4_ or S, P-g-C_3_N_4_ powder were suspended in 30 mL water and ultrasonicated for 10 min, followed by the dropwise addition of Zn(NO_3_)_2_ and Cr(NO_3_)_3_ solutions (2:1 molar ratio). By introducing 1 M NaOH, the mixture’s pH was carefully regulated within the 7–8 range, and 1 M NaHCO_3_ was added for CO_3_^2−^ intercalation, then stirred, washed, and dried. The g-C_3_N_4_ did not intercalate into the LDHs interlayer (which was occupied by intercalated CO_3_^2−^) but instead preferentially adheres to the external surfaces of in situ formed ZnCr-LDH nanosheets through electrostatic interactions, serving as a 2D substrate to support the in situ growth of LDHs. This conclusion was corroborated by the results of characterization techniques including X-ray diffraction (XRD), X-ray photoelectron spectroscopy (XPS), and Transmission electron microscopy (TEM). This process maximized heterojunction advantages, enhancing interfacial charge migration and catalytic activity. Under visible-light conditions, the composite exhibited the excellent ability to produce hydrogen and degrade ciprofloxacin simultaneously, with good stability, demonstrating the superiority of the in situ co-precipitation method in constructing hierarchical heterostructures.

**Figure 4:**
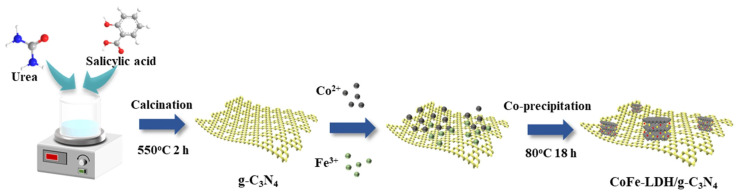
The synthetic process diagram of CoFe-LDH/g-C_3_N_4_. The blue small spheres represent nitrogen atoms (N), and the red small spheres represent oxygen atoms (O), while the unmarked (grey/white) spheres correspond to carbon (C) and hydrogen (H) atoms, respectively. Green small particles stand for Fe^3+^ ions, black small particles represent Co^2+^ ions. The yellow grid-like framework corresponds to the g-C_3_N_4_, the gray layered regions represent the layered structural units of CoFe-LDH. Reprinted with permission from Ref. [49].

Li et al. [49] fabricated a CoFe-LDH/g-C_3_N_4_ nanocomposite featuring a prominent heterostructure through facile co-precipitation (Figure 4). The synthesis involved creating an aqueous suspension containing g-C_3_N_4_ along with Co(NO_3_)_2_·6H_2_O and Fe(NO_3_)_3_·9H_2_O precursors. CoFe-LDH nanosheets uniformly loaded onto g-C_3_N_4_ surfaces through the co-precipitation method, forming intimate interfacial contacts. This structure provided additional active centers as well as shorter charge migration paths, significantly improving photogenerated carrier separation efficiency. Compared to individual g-C_3_N_4_ and pristine CoFe-LDH, this composite showed improved catalytic performance in the degradation of tetracycline, along with good stability and practical potential.

#### 3.1.3. Hydrothermal/Solvothermal Method

The hydrothermal/solvothermal technique involves chemical reactions in a sealed reactor (e.g., autoclave) with water or organic solvents employed as the reaction medium under high temperature (typically 100–250 °C) and autogenous pressure [66]. Typically, g-C_3_N_4_ precursors (e.g., melamine) and LDH precursors (metal salts), or pre-synthesized g-C_3_N_4_ and LDH nanomaterials, are dispersed in water (hydrothermal) or organic solvents (solvothermal), sealed in the reactor, and reacted at a specific temperature (typically 100–200 °C) under autogenous pressure [67]. The high-temperature and high-pressure environment facilitates material crystallization and in situ heterojunction formation, yielding composites with high crystallinity, controllable morphology, and robust interfacial contact that enhances charge transfer [68].

**Figure 5:**
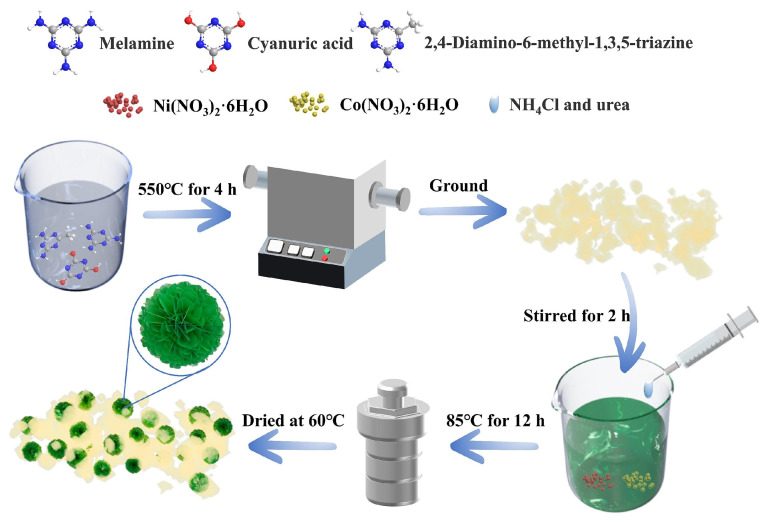
The synthesis diagram of NiCo-LDH/g-C_3_N_4_. The blue small spheres represent nitrogen atoms (N), and the red small spheres represent oxygen atoms (O), the unmarked (grey/white) spheres correspond to carbon (C) and hydrogen (H) atoms, respectively. Yellow/white particles (after grinding) are the g-C_3_N_4_-based precursor obtained via calcination. Green solution in the right beaker is the composite dispersion system, green spherical structure (enlarged view) represents the flower-like NiCo-LDH. Reprinted with permission from Ref. [69].

For example, Gu et al. [69] prepared NiCo-LDH/g-C_3_N_4_ (NCH/SCN) composites with varying mass ratios via in situ hydrothermal growth (Figure 5). Using aqueous medium to dissolve nickel nitrate hexahydrate, cobalt nitrate hexahydrate, ammonium chloride, urea, and smoke-like g-C_3_N_4_ (SCN), the mixture underwent hydrothermal treatment to achieve interfacial integration. This process promoted tight integration between the porous SCN and flower-like NiCo-LDH, forming a Z-Scheme heterojunction. The optimized NCH/SCN exhibited strong photoresponse and optimal hydrogen evolution (3125 μmol·g^−1^·h^−1^ at 420 nm), attributed to reduced charge-transfer distance and an internal electric field. The catalyst maintained high activity after five cycles.

Srisuvetha et al. [70] fabricated mesoporous g-C_3_N_4_/CoAl-LDH composites via one-step hydrothermal reaction to realize efficient hydrogen evolution via photocatalysis. A homogeneous precursor was formed by mixing Co(NO_3_)_2_·6H_2_O, Al(NO_3_)_3_·9H_2_O, NH_4_F and urea solutions under magnetic stirring, and then g-C_3_N_4_ dispersion solution was added. The reaction was conducted at 160 °C with a reaction duration of 2 h. CoAl-LDH nanosheets grew uniformly at g-C_3_N_4_ surfaces through crystallization processes, forming a close interfacial contact. This structure shortened the charge transfer distance via electrostatic interactions and hydrogen bonds, which suppressed electron-hole recombination. The composite showed exceptional hydrogen production (3677.5 μmol·h^−1^·g^−1^), representing a 7.5-fold enhancement compared to pristine CoAl-LDH (490.5 μmol·h^−1^·g^−1^).

#### 3.1.4. Physical or Mechanical Mixing Method

The physical or mechanical mixing method represents the simplest and most direct preparation approach. It typically involves the mechanical mixing of pre-synthesized g-C_3_N_4_ powder and LDHs powder in either the solid phase (e.g., ball milling) or liquid phase (e.g., ultrasonic dispersion followed by solvent evaporation) [71]. This method offers exceptional operational simplicity, low cost-effectiveness, along with good adaptability for large-scale manufacturing.

**Figure 6:**
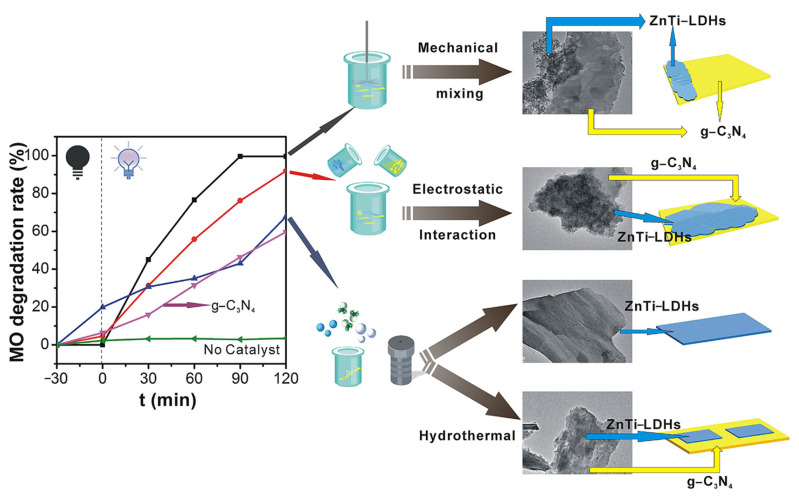
Comparison diagram of the three preparation methods. In the left figure, black curve corresponds to the MO degradation curve of the composite prepared by the mechanical mixing method; red curve represents the MO degradation curve of the composite obtained via the electrostatic self-assembly method; blue curve stands for the MO degradation curve of the composite synthesized by the hydrothermal method; purple curve (labeled “g-C_3_N_4_”) denotes the MO degradation efficiency curve of pure graphitic carbon nitride (g-C_3_N_4_); green curve (labeled “No Catalyst”) indicates the MO degradation curve under the same light conditions without any catalyst. On the right side, Yellow structural motifs represent g-C_3_N_4_, blue structural motifs correspond to ZnTi-LDHs; On the top of the third beaker in the middle, blue small spheres represent zinc ions; gray small spheres represent titanium ions; the molecular structure in the middle represent urea. Reprinted with permission from Ref. [72].

For instance, Zhang et al. [72] fabricated ZnTi-LDH/g-C_3_N_4_ composites through a straightforward physical blending approach to realize efficient degradation of methyl orange(MO) via photocatalysis. Pre-synthesized ZnTi-LDH and g-C_3_N_4_ were ground in a mortar for 10 min, suspended in 20 mL aqueous medium, and underwent 24 h agitation under ambient conditions. During this process, mechanical shear forces facilitated the uniform dispersion and intimate contact between ZnTi-LDH nanosheets and g-C_3_N_4_ layers, forming a “sheet-to-sheet” structure. For comparison, composites were also fabricated via hydrothermal approaches and electrostatic self-assembly to investigate how synthesis means affect morphology, structure, and photocatalytic activity (Figure 6). Results demonstrated that the mechanically mixed ZnTi-LDH/g-C_3_N_4_ composite exhibited superior photocatalytic performance for MO degradation. The improved activity stemmed from the development of a hierarchical “layer-on-layer” architecture between g-C_3_N_4_ and ZnTi-LDH components. The distinctive architecture of this composite effectively facilitated the segregation of photoinduced charge carriers, substantially boosting its photocatalytic performance. This study proved that simple physical mixing enabled efficient construction of 2D/2D heterojunctions without complex equipment or high-temperature/high-pressure conditions, offering a novel strategy for designing high-performance photocatalysts suitable for large-scale production.

Sherryna et al. [73] prepared xNiCoAlCN composites with varying mass ratios via physical mixing. Synthesized g-C_3_N_4_ (gCN) was first dispersed in methanol under continuous stirring for 2 h to ensure particle dispersion. Ni_x_Co_y_Al_z_-LDH with different cation compositions was then added to the solution, followed by another 2 h of stirring, and final drying in an oven. This method was simple, avoided high temperatures or chemical modification steps, and reduced cost and time. After physical mixing, NiCoAl-LDH nanosheets were well-dispersed on the gCN planar substrate, forming intimate interfacial contact and maximizing active site exposure. This facilitated an S-Scheme charge transfer mechanism, significantly enhancing hydrogen generation performance.

#### 3.1.5. Calcination Method

Calcination is commonly employed as a post-treatment step or a direct synthesis approach. Three calcination methods exist [56]: (i) direct calcination of pre-synthesized g-C_3_N_4_/LDHs composites; (ii) initial calcination of LDHs to generate mixed metal oxides (MMOs), followed by calcination of the MMO and g-C_3_N_4_ mixture; and (iii) co-calcination of the raw mixture of LDHs and g-C_3_N_4_ precursors. The g-C_3_N_4_ exhibits excellent thermal stability with a thermal decomposition temperature at about 600 °C [74]. Meanwhile LDHs only completely decompose into composite metal oxides (LDO) at temperatures above 400–500 °C. Therefore, in the composite process, a calcination temperature range of 450–550 °C is typically employed, within which g-C_3_N_4_ can retain its inherent layered structure and photocatalytic activity without significant oxidation or decomposition [75]. Air is the most commonly used atmosphere (owing to its simplicity and cost-effectiveness), while an inert atmosphere (such as N_2_ or Ar) is preferred to avoid potential oxidative degradation of g-C_3_N_4_ at higher temperatures [76].

**Figure 7:**
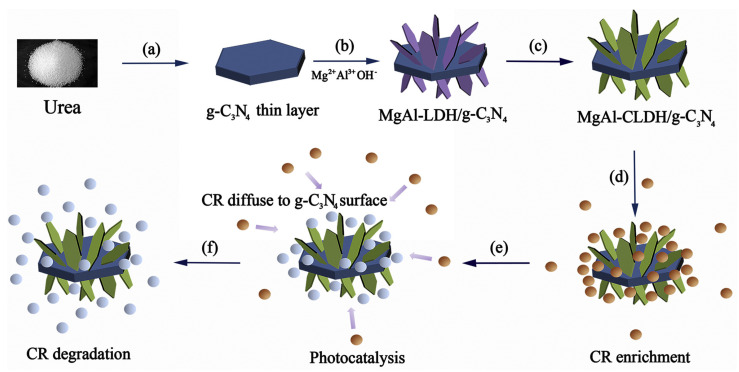
The synthesis diagram of MgAl-CLDH/g-C_3_N_4_. (**a**) g-C_3_N_4_ preparation: Urea thermally polymerizes into layered g-C_3_N_4_ thin sheets. (**b**) LDH growth: Mg/Al ions assemble into MgAl-LDH nanosheets on g-C_3_N_4_ via co-precipitation. (**c**) LDH→CLDH transformation: Calcination decomposes MgAl-LDH into amorphous MgAl-CLDH. (**d**) CR enrichment: MgAl-CLDH electrostatically adsorbs anionic CR. (**e**) Photocatalysis preparation: CR diffuses to g-C_3_N_4_; light excites g-C_3_N_4_ to generate charge carriers. (**f**) CR degradation: Photogenerated active species oxidize CR into small-molecule products. In the figure, dark blue polygonal structure corresponds to the g-C_3_N_4_ thin layer; Purple acicular structure stands for the MgAl-LDH; Green acicular structure denotes the MgAl-CLDH; Brown small spheres represent the undegraded contaminant CR; Gray small spheres stand for the small-molecule products generated after the contaminant CR (originally brown small spheres) is photocatalytically degraded. Reprinted with permission from Ref. [77].

For example, Wang et al. [77] synthesized MgAl-CLDH/g-C_3_N_4_ composites via coprecipitation and calcination (Figure 7). They first prepared g-C_3_N_4_ thin films via urea thermal polymerization, then used g-C_3_N_4_ nanosheets as a growth scaffold to trigger vertical-oriented growth of MgAl-LDH. Subsequent calcination of the mixture was conducted at 400 °C for 1 h in air, which facilitated in situ formation of nanoscale mixed oxides within MgAl-LDH nanosheets, yielding freely standing MgAl-LDH on g-C_3_N_4_ nanosheets. XRD pattern confirmed that after being calcined at 400 °C, the g-C_3_N_4_ component remained structurally stable and no oxidation decomposition was observed. The calcined composites exhibited a reduced band gap (2.63 eV), broader visible-light absorption range, as well as enhanced photocatalytic efficiency. XPS analysis revealed improved electron transfer and delocalization effects, which suppressed photogenerated carrier recombination, thereby enhancing photocatalytic functionality. This treatment demonstrated an effective adsorption–desorption synergy under simulated sunlight, enabling efficient removal of the anionic pollutant congo red (CR) from water.

#### 3.1.6. Other Synthetic Methods

Beyond mainstream approaches, novel techniques have been adopted to construct high-efficiency g-C_3_N_4_/LDHs composites for finer structural control, including the coating method [78], the microwave irradiation method [79], the impregnation method [80], and the polydopamine cross-linking method [81], etc.

For instance, Alam et al. [82] developed a Ag-doped g-C_3_N_4_@NiFe-LDH composite through a spin disk reactor (SDR) aiming at the photodegradation of rhodamine B (RhB). Ni/Fe nitrates and NaOH were mixed in the SDR under high rotation, forming uniform nanoparticles, followed by hydrothermal treatment (120 °C, 6 h) for crystallization. The g-C_3_N_4_ nanosheets were dispersed into metal salt solutions for uniform compositing via SDR, and Ag nanoparticles were photodeposited. The SDR-synthesized NiFe-LDH exhibited a 3D flower-like structure, forming tight heterojunctions with g-C_3_N_4_, narrow particle distribution, and high crystallinity, increasing active sites and surface area. The Ag doping and Z-Scheme heterojunction enhanced photocatalytic activity, achieving 99% RhB degradation in 240 min.

**Figure 8:**
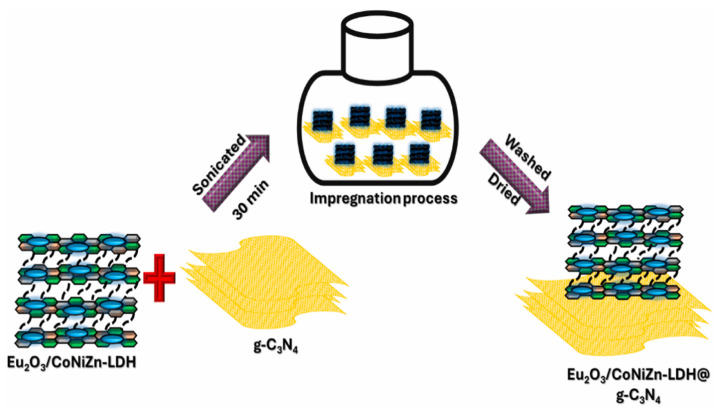
The synthesis diagram of Eu_2_O_3_@CoNiZn-LDH/g-C_3_N_4_. Green/orange layered structures (left) represent CoNiZn-LDH, the blue elliptical particles (on LDH layers) stand for Eu_2_O_3_ nanoparticles (the “Eu_2_O_3_@” component in the composite); Yellow flake-like structures correspond to g-C_3_N_4_. Reprinted with permission from Ref. [83].

Maridevaru et al. [83] prepared Eu_2_O_3_@CoNiZn-LDH/g-C_3_N_4_ (LDHCN) composites via a simple impregnation method for solar-driven H_2_ production (Figure 8). LDH nanosheets and g-C_3_N_4_ were mixed at varying ratios, ultrasonicated, and dried. The impregnation method ensured uniform LDH dispersion on g-C_3_N_4_, forming intimate heterojunctions that broadened light absorption and provided active sites. This simple, scalable method enabled performance optimization by tuning LDH loading, providing a new idea for designing efficient hydrogen-producing catalysts.

#### 3.1.7. Critical Comparison on Preparation Methods

The hydrothermal method excels in crystallinity and interfacial robustness, making it suitable for high-performance laboratory-scale catalysts, but its reliance on autoclaves leads to high energy consumption and poor scalability. In contrast, electrostatic self-assembly operates under ambient conditions, requires simple equipment, and is easily scaled up for industrial production, though interfacial interactions are dominated by electrostatic forces (weaker than chemical bonds formed via hydrothermal treatment). Co-precipitation enables in situ LDHs growth on g-C_3_N_4_, ensuring uniform dispersion and tight contact, but requires precise pH control. Physical mixing is the simplest and cheapest method for bulk production, but suffers from uneven dispersion and weak interfacial synergy, limiting its application in high-demand scenarios. Calcination improves the stability of composites and modifies band structure to enhance photocatalytic activity, but excessive temperature (>550 °C) may cause g-C_3_N_4_ oxidation or LDH structural collapse. For industrial applications, methods with low energy input, simple operation, and easy scale-up (e.g., physical mixing or self-assembly) are attractive, while research-oriented studies often prioritize optimal interfacial engineering via hydrothermal or co-precipitation routes. Table 1 compares their pros and cons, as well as their stability, cost, and scalability.

### 3.2. Performance Optimization Strategies

Beyond selecting appropriate synthesis methods, the g-C_3_N_4_/LDHs composites’ photocatalytic performance is capable of being further boosted via multiple fine-tuning strategies. These strategies directly regulate the materials’ band gap structure and catalytic activity by modifying synthesis parameters, structural features, and interfacial interactions.

#### 3.2.1. Ratio Control

The mass or molar ratio of g-C_3_N_4_ to LDHs is a critical parameter, directly influencing light absorption, heterojunction interface quality, charge separation efficiency, and active site density [84]. An excessively low ratio yields insufficient synergistic effects, while an overly high ratio may induce a “shielding effect” (where one component obstructs light absorption/reactant contact) or increase charge recombination centers [85]. Thus, an optimal ratio balances these factors for peak performance. More importantly, the ratio determines the degree of interfacial coupling between g-C_3_N_4_ and LDHs, thereby regulating the band gap of the composite.

**Figure 9:**
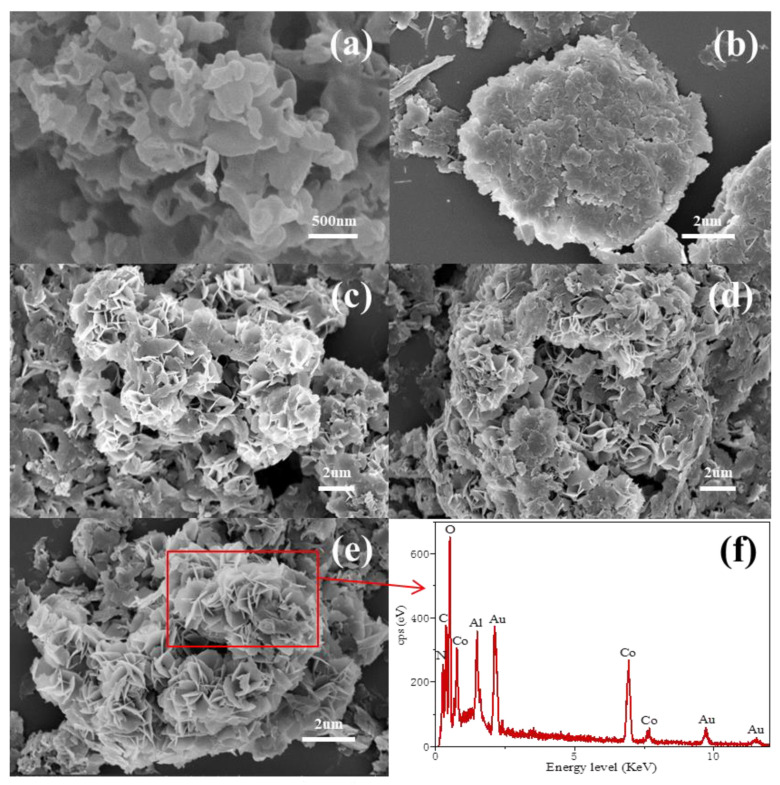
SEM micrographs of: (**a**) pure g-C_3_N_4_, (**b**) CoAl-LDH, (**c**) 0.75 wt% CoAl-LDH-modified g-C_3_N_4_ composite, (**d**) 1.25 wt% CoAl-LDH-modified g-C_3_N_4_ composite, (**e**) 1.5 wt% CoAl-LDH-modified g-C_3_N_4_ composite, (**f**) EDS of 1.5 wt% g-C_3_N_4_/CoAl-LDH composite. Reprinted with permission from Ref. [86].

For example, Huang and colleagues [86] fabricated g-C_3_N_4_/CoAl-LDH composites with unique nanoflower structures at varying g-C_3_N_4_ proportions (denoted as g-C_3_N_4_/CoAl-LDH0.75, g-C_3_N_4_/CoAl-LDH1.25, and g-C_3_N_4_/CoAl-LDH1.5). SEM analysis (Figure 9) revealed that the microstructure of the composites was influenced by the g-C_3_N_4_ content, with distinct morphological variations observed. The g-C_3_N_4_/CoAl-LDH1.5 composite exhibited the maximum crystallinity as well as most uniform particle size, forming well-defined nanoflower structures. Concurrently, the findings indicated that the NO_x_ removal efficiency was substantially improved as the g-C_3_N_4_ loading increased. The underlying reason for this lied in the introduction of g-C_3_N_4_ effectively suppressing interlayer agglomeration in CoAl-LDH, thereby enhancing interfacial charge transfer and narrowed the composite’s band gap (from ~2.66 eV of pure g-C_3_N_4_ to ~2.49 eV), broadening visible-light absorption. The specific surface area increased to 70 m^2^/g and the narrowed band gap promoted photogenerated carrier separation, leading to significantly improved NO_x_ removal efficiency. It can be observed that adjusting the feed ratio allows for the identification of an equilibrium point to achieve optimal photocatalytic performance in the composite material.

Zhang et al. [87] fabricated CoFe-LDH/g-C_3_N_4_ composite photocatalysts at varying g-C_3_N_4_ content (1.5%, 3.0%, 4.5%, 6%) via a hydrothermal process. The band gap first narrowed and then widened with increasing g-C_3_N_4_ content: the 3% g-C_3_N_4_ sample exhibited the smallest band gap (~2.05 eV) and peak tetracycline (TC) degradation efficiency (94.98%). Increasing the g-C_3_N_4_ content further reduced the efficiency, owing to weakened interfacial coupling and a widened band gap. Additionally, the Co^2+^/Fe^3+^ ratio in CoFe-LDH was optimized, revealing that TC removal efficiency improved with higher Co^2+^ content, achieving optimal performance (94.8%) at a Co^2+^:Fe^3+^ ratio of 3:1. This illustrated that fine-tuning component ratios can identify a “peak performance point”.

#### 3.2.2. Morphology Design

The macroscopic and microscopic morphology of materials (e.g., dimension, size, pore structure, assembly pattern) profoundly influences their specific surface area, light-harvesting capability, mass transfer, and interfacial contact [88]. Careful morphology design enables the construction of structures with enhanced catalytic activity. Meanwhile, the special morphology can narrow the band gap, enhance the interfacial interaction between components, and promote electron transfer, thereby increasing the specific surface area and enhancing the light absorption efficiency.

**Figure 10:**
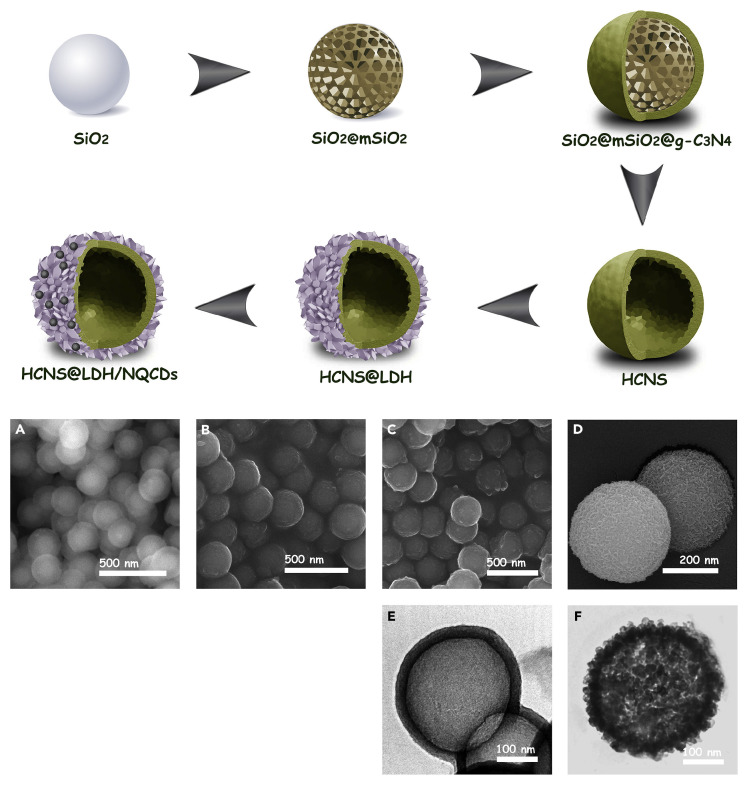
HCNS@LDH/NCQDs: preparation and morphological characterization (FESEM/TEM): (**A**) SiO_2_@mSiO_2_ (FESEM), (**B**) SiO_2_@mSiO_2_@g-C_3_N_4_ (FESEM), (**C**) HCNS (FESEM), (**D**) HCNS@LDH/NCQDs (FESEM), (**E**) HCNS (TEM), (**F**) HCNS@LDH/NCQDs (TEM). In the figure, white solid spheres represent the silica template (SiO_2_). Golden core-shell spheres correspond to the mesoporous silica-coated silica (SiO_2_@mSiO_2_) structure. Green core-shell spheres stand for the SiO_2_@mSiO_2_@g-C_3_N_4_, where the green outer layer denotes g-C_3_N_4_. Green hollow spheres represent hollow carbon nitride spheres (HCNS), obtained after removal of the SiO_2_ template. Purple-coated hollow spheres correspond to the HCNS@LDH composite, in which the purple layer refers to CoAl-LDH. Purple hollow spheres with bright gray spherical particles represent the target HCNS@LDH/NCQDs composite, where the bright gray spherical particles denote nitrogen-doped carbon quantum dots (NCQDs). Reprinted with permission from Ref. [89].

For instance, Arjomandi-Behzad et al. [89] engineered a core–shell architecture (Figure 10), where hollow carbon nitride spheres (HCNS) served as structural supports, while CoAl-LDH modified by nitrogen-doped carbon quantum dots (NCQDs) constituting the outer layer for enhanced visible-light photocatalytic performance. This method first prepared hollow HCNS using SiO_2_ as a template, followed by solvothermal deposition of LDH and NCQDs on HCNS to form a ternary heterojunction. The optimized interfacial integration between the LDH shuck and g-C_3_N_4_ kernel facilitated high-efficiency photogenerated charge separation and induced band gap narrowing (from 2.74 eV of pure g-C_3_N_4_ to 2.48 eV), enhancing visible-light absorption. Owing to the hollow porous architecture, tight interfacial connections, extensive specific surface area, and synergistic effects among components, the engineered nanocomposite demonstrated superior photocatalytic efficiency and microbial inactivation capabilities. This study pioneered new pathways for tailoring high-performance photochemical nanomaterials.

#### 3.2.3. Defect Engineering

The introduction of appropriate defects, such as atomic vacancies and interstitial atoms, into semiconductors serves as an effective strategy for band structure modulation [90]. Defect incorporation can generate additional energy levels inside the band gap and reduce the effective bandgap, thereby broadening the light response range, acting as carrier traps to promote charge separation, or directly functioning as catalytic active centers [91].

For instance, Zheng and colleagues [92] fabricated a layered g-C_3_N_4_/LDH composite (g-C_3_N_4_/LDH-OVs) rich in oxygen vacancies. The visible-light-driven photocatalytic system demonstrated effective photocatalytic breakdown and thorough mineralization of tetracycline hydrochloride (TC). Defect formation was achieved through structural induction strategy: pristine LDH powder was mixed with ethylene glycol and sodium hydroxide, reacted at 140 °C for 12 h—thermal treatment-induced lattice distortion and hydroxyl loss during this process generated abundant oxygen vacancies (OVs). The existence of oxygen vacancies was verified by multiple characterization techniques: XPS showed a characteristic peak at 531.5 eV in the O 1s spectrum corresponding to defect oxygen, and electron paramagnetic resonance (EPR) detected a distinct signal at g = 1.973, confirming OVs enrichment. The introduced oxygen vacancies not merely furnished plentiful active centers but also served as charge-carrier reservoirs, restraining the combination of photogenerated carriers as well as boosting carrier separation efficiency—DFT calculations confirmed that OVs formed new defect levels, narrowing the band gap by 9.0 eV and enhancing electronic conductivity. Experimental data confirmed that the optimized photocatalytic material achieved degradation and mineralization rates of 95% and 28%, respectively, following 60 min of visible-light illumination, which corresponded to 2.3-fold and 3.5-fold increases relative to bare g-C_3_N_4_ and LDH-OVs.

**Figure 11:**
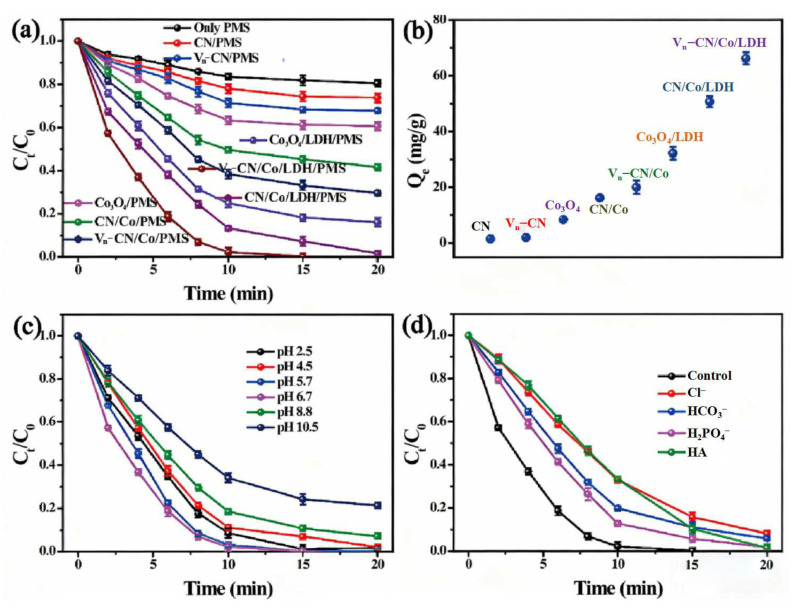
(**a**) Catalyst degradation efficiency, (**b**) adsorption efficiency, (**c**) pH dependence, (**d**) effects of inorganic ions and natural organic matter. Reprinted with permission from Ref. [93].

Yang et al. [93] developed a super-hydrophilic nitrogen/oxygen dual-defect enriched g-C_3_N_4_/LDH heterostructure composite (Vn-CN/Co/LDH) for effective peroxymonosulfate (PMS) activation in organic pollutant degradation, particularly ofloxacin (OFX), achieving ~100% OFX degradation in 15 min (Figure 11a). Initially, nitrogen vacancies were introduced into g-C_3_N_4_ nanosheets via NaBH_4_ reduction to obtain nitrogen vacancy-rich Vn-CN substrates. Cobalt-modified Vn-CN/Co was then synthesized using a heterogeneous nucleation approach assisted by low concentration, and subsequent in situ growth of CoAl-LDH on Vn-CN/Co via coprecipitation to form a two-dimensional heterostructure with nitrogen/oxygen dual vacancies. Defect presence was validated by XPS (N 1s binding energy shift due to electron redistribution, O 1s peak at 532.4 eV assigned to oxygen vacancies) and EPR (enhanced signal intensity relative to pristine g-C_3_N_4_). Results indicated that the nitrogen and oxygen vacancies exposed more active sites and significantly enhanced superhydrophilicity, achieving an OFX adsorption capacity of 63.3 mg/g (Figure 11b), far exceeding conventional catalysts. Moreover, it maintained >80% degradation at pH 2.5–10.5, adapting to real wastewater without pH adjustment (Figure 11c). Only slight efficiency declined with ions/organic matter, benefiting from radical-nonradical synergy (Figure 11d). The multi-vacancy structure modulated the electronic structure, reduced charge transfer resistance, enabling high-efficiency pollutant degradation through defect engineering and interface modulation strategies.

#### 3.2.4. Elemental Doping

Elemental doping entails incorporating trace amounts of foreign elements (metals or non-metals) to the lattice of g-C_3_N_4_ or LDHs to substitute for original C/N or metal atoms, thereby modifying their electronic structure, conductivity, and optical properties [94]. For instance, doping transition metals for instance Cu^2+^, Fe^3+^, and Zn^2+^ to the g-C_3_N_4_ lattice effectively reduces its band gap [95], enhancing photocatalytic performance. Similarly, non-metallic element doping (e.g., P, S, O, or B) regulates g-C_3_N_4_ properties, improving photocurrent response and carrier separation performance through incorporating new energy levels and narrowing the band gap [96].

Bilal et al. [97] prepared a high-performance sulfur-doped S-Scheme heterojunction composite (Mxene/S-g-C_3_N_4_/NiAl-LDH) for sustainable photocatalytic hydrogen production. Initially, NiAl-LDH nanosheets were synthesized via hydrothermal method, followed by sulfur doping through thermal decomposition of thiourea to yield sulfur-doped g-C_3_N_4_, with subsequent nitric acid treatment to enhance surface activity. Finally, a ternary composite was hydrothermally constructed by optimizing the S-g-C_3_N_4_ and Mxene loading ratios, forming an intimate S-Scheme heterojunction interface. Sulfur doping played a critical role in g-C_3_N_4_, reducing its band gap narrowed spanning 2.78 eV to 2.65 eV, broadening visible-light utilization efficiency, increasing specific surface area, and enhancing the efficiency of photogenerated charge separation. This optimization also shifted the conduction band position negatively, boosting reducing ability and photocatalytic hydrogen production activity.

**Figure 12:**
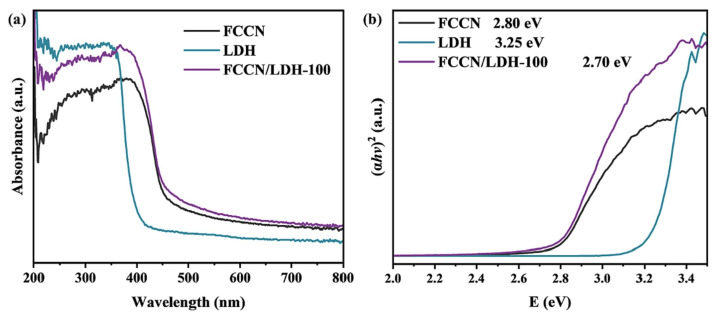
(**a**) UV-vis DRS, (**b**) (αhv)^2^ vs. photon energy for FCCN, LDH, and FCCN/LDH-100. Reprinted with permission from Ref. [98].

Hu et al. [98] developed a novel composite photocatalyst through halogen (F, Cl) doping in g-C_3_N_4_ combined with ZnAl-LDH (FCCN/LDH) for efficient tetracycline (TC) degradation in seawater. F and Cl co-doped g-C_3_N_4_ (FCCN) was fabricated through thermal polymerization of melamine with NH_4_F and NH_4_Cl, then composited with LDH through coprecipitation to form an intimate 2D/2D heterostructure. As shown in Figure 12b, halogen atom incorporation optimized the g-C_3_N_4_ band structure, reducing band gap energy (spanning 2.80 eV to 2.70 eV), increasing charge carrier mobility, suppressing electron-hole recombination, and broadening visible-light response via absorbing edge red shift (Figure 12a). This composite showed exceptional photocatalytic degradation under visible light, with TC efficiency reaching 95.5%, significantly outperforming individual FCCN and LDH materials.

#### 3.2.5. Constructing Multinary Heterojunctions

Building upon g-C_3_N_4_/LDHs binary composites, the introduction of third or even fourth components—such as noble metal nanoparticles, other semiconductors, and carbon materials (graphene, carbon dots)—to construct ternary or multinary heterojunctions represents an effective strategy for further enhancing performance [99]. Heterojunctions can optimize the energy band alignment and narrow the effective band gap, while retaining strong redox capabilities and enhancing the photocatalytic degradation efficiency.

To illustrate Kaur and colleagues [100] developed Au/LDH/g-C_3_N_4_ ternary nanocomposites. Initially, NiAl-LDH with a loading amount of 5–15 wt% was loaded on g-C_3_N_4_ (CN) via self-assembly technology to form NiAl-LDH/CN composites, followed by photochemical deposition of Au nanoparticles. The tight “face-to-face” interface between NiAl-LDH and g-C_3_N_4_ shortened charge-transfer distances, while Au nanoparticles enhanced visible-light utilization efficiency via surface plasmon resonance (SPR). The band alignment of the ternary heterojunction enabled a stepwise electron migrate pathway from g-C_3_N_4_ to NiAl-LDH and then to Au, narrowing the composite’s band gap and retaining strong redox capacity, effectively separating charges and prolonging carrier lifetimes, thereby boosting catalytic activity. Under visible light, this ternary composite achieved 97.14% degradation efficiency for tetracycline (TCH), significantly outperforming single or binary components.

Niknam et al. [101] hydrothermally developed a g-C_3_N_4_/NiAl-LDH/CeO_2_ ternary composite for efficient rhodamine B (RB) degradation. NiAl-LDH bridged g-C_3_N_4_ and CeO_2_ via electrostatic interactions and chemical bonding, forming stable interfaces that facilitated electron transfer. The Z-Scheme heterojunction optimized band alignment, narrowing the composite’s band gap to ~2.54 eV (vs. 2.68 eV of g-C_3_N_4_) and broadening light absorption to UV-visible regions. The Z-Scheme mechanism combined with synergism among g-C_3_N_4_, LDH, as well as CeO_2_ boosted light utilization efficiency, charge separation, and catalytic activity. This ternary composite degraded 98% of RB under UV light in 350 min, with broadened absorption (UV to visible light). Multinary heterojunctions optimized charge separation, light harvesting, and interfacial interactions, demonstrating significant potential for environmental remediation.

## 4. Mechanisms of Photocatalytic Degradation of Water Pollutants

A deep understanding of the photodegradation mechanisms of pollutants by g-C_3_N_4_/LDHs composite materials is crucial for directing the development of more highly effective catalysts, with the core principles lying in effective charge separation as well as the production of highly active species [102]. In this section, three main dominant core mechanisms governing the photocatalytic decomposition of water contaminants by g-C_3_N_4_/LDHs composite materials are selected and systematically summarized: heterojunction charge separation mechanisms (including Type-II, Z-Scheme, and S-Scheme), synergistic adsorption-photocatalysis mechanisms, and persulfate-assisted photocatalytic mechanisms.

### 4.1. Heterojunction Charge Separation Mechanisms

Constructing heterojunctions by intimate contact between two or more semiconductors provide a pivotal means to boost photocatalytic activity [103]. By regulating the band structures of different semiconductor materials, specific built-in electric fields and charge transfer pathways can be established, effectively minimizing electron-hole pair recombination while prolonging carrier lifetimes [104]. In light of the distinct band alignment characteristics of g-C_3_N_4_ and LDHs, the primary heterojunction mechanisms in g-C_3_N_4_/LDHs systems involve Type-II, Z-Scheme, and S-Scheme heterojunctions [105].

#### 4.1.1. Type-II Heterojunction

This is the most traditional and classical heterojunction model. In this model, both the conduction band (CB) and valence band (VB) potentials of g-C_3_N_4_ are more negative compared to LDHs, resulting in a straddling band alignment between the two semiconductors [84]. The relative band potentials create favorable conditions where photoexcited electrons in g-C_3_N_4_’s conduction band become thermodynamically favored to flow towards the lower-energy LDH conduction band, while photogenerated holes in LDH’s valence band are simultaneously transported to the elevated valence band of g-C_3_N_4_ [83]. This process achieves effective separation of photogenerated electrons and holes—electrons accumulate on LDH, and holes accumulate on g-C_3_N_4_ [106]. These separated electrons and holes then undergo redox reactions with surface-adsorbed O_2_ and H_2_O, thereby forming reactive species including ·O_2_^−^, ·OH, which subsequently degrade pollutants [107].

For example, Maridevaru et al. [108] prepared a Type-II CoAl-LDH@g-C_3_N_4_ (CACN) composite through a straightforward impregnation method to realize photocatalytic H_2_ production and photodegradation of dye pollutants. This material, based on CoAl-LDH and g-C_3_N_4_, formed a 2D/2D heterostructure through electrostatic self-assembly. The engineered Type-II configuration remarkably inhibited charge carrier recombination. Through band alignment, electrons migrated from CoAl-LDH’s conduction band (CB) toward g-C_3_N_4_’s valence band (VB), recombining with the VB holes of g-C_3_N_4_. The retained CB electrons in g-C_3_N_4_ reduced adsorbed O_2_ to ·O_2_^−^ and can also facilitate ·OH formation through water interaction, whereas CoAl-LDH’s VB holes induced the oxidation of H_2_O to yield ·OH. These ROS possessed strong oxidizing ability, enabling degradation of dye compounds into harmless inorganic species such as CO_2_ and H_2_O. The results revealed that the 5 wt% CoAl-LDH-loaded composite(5-CACN) displayed the outstanding photocatalytic performance under visible light, the system attained a 79% decomposition efficiency for Brilliant Black (BN) dye, demonstrating excellent catalytic performance.

Salehi et al. [48] fabricated a novel ternary heterojunction MgAl-LDH@g-C_3_N_4_@Ag_3_PO_4_ composite materials to enhance the photodegradation efficiency for methylene blue (MB) dye degradation. The synergistic effects within the ternary system optimized light absorption efficiency and facilitated charge carrier mobility via strategic band structure alignment, substantially boosting catalytic activity. Experimental data indicated that the catalyst achieved 99% MB degradation efficiency within 45 min, and a Type-II heterojunction mechanism for the degradation process was put forward. Since the CB level of Ag_3_PO_4_ was closer to the VB level of g-C_3_N_4_, photoinduced electrons from silver phosphate recombined with g-C_3_N_4_’s vacancies while residual holes(h^+^) in Ag_3_PO_4_’s valence band combined with H_2_O to yield ·OH. On account of the CB of g-C_3_N_4_ exhibited a lower conduction band potential (−1.16 V), electrons migrated from this CB to that of MgAl-LDH, reacting with O_2_ to form ·O_2_^−^. The generated reactive species (·O_2_^−^, ·OH, and h^+^) underwent redox reactions with MB molecules, gradually degrading them into CO_2_ and H_2_O. Experiments confirmed that photogenerated holes and ·OH served as the primary reactive species, whereas ·O_2_^−^ played a relatively weaker role.

#### 4.1.2. Z-Scheme Heterojunction

The Type-II heterojunction offers a unique advantage of a clear mechanism, enabling intuitive spatial separation of photogenerated charge carriers across different semiconductors, which significantly decreases recombination probability [109]. However, its drawback is that the separated electrons (e^−^) and h^+^ occupy energy levels with relatively weak redox capabilities (e.g., LDH’s CB and g-C_3_N_4_’s VB), leading to a diminished overall oxidation capacity compared to the strongest capabilities of individual components [110]. This “sacrifice of redox capability for charge separation” model results in weakened oxidative properties of active species, potentially failing to degrade structurally stable and recalcitrant organic pollutants effectively [111]. To address these drawbacks, researchers developed the Z-Scheme charge transfer mechanism. In this model, conduction band electrons from g-C_3_N_4_ directly recombine with valence band holes in LDH, confining electrons in the more reducing LDH’s CB (if LDH’s CB potential is more negative), while there were holes in the more oxidizing g-C_3_N_4_ valence band [112]. This achieves efficient charge separation and maintains superior redox potential for pollutant degradation [113].

Hu et al. [114] developed a sulfurized ZnAl LDH (ZAS)-modified g-C_3_N_4_ composite, ZnAlSx@g-C_3_N_4_(ZASCN), via hydrothermal methods to achieve highly effective photodegradation of tetracycline (TC). Results showed that ZASCN-3 (30% ZAS mass fraction) achieved 94.05% TC degradation within 180 min. This material combined ZAS’s sulfide properties and g-C_3_N_4_’s visible-light response, forming tight interfacial interaction and a high-efficiency Z-Scheme charge separation system. The interfacial charge transfer facilitated merging of ZAS’s conduction band electrons with g-C_3_N_4_’s valence band holes, thereby preserving the oxidative potential of ZAS’s valence band holes while maintaining the reductive capacity of g-C_3_N_4_’s conduction electrons. This transfer path effectively inhibited direct recombination of electron-hole pairs, prolonged the carrier lifetime, and simultaneously retained the charges with high redox potentials in both materials. The holes on the VB of ZAS had strong oxidizing capacity and were capable of oxidizing dye molecules directly or reacting with water molecules to produce ·OH. Concurrently, conduction band electrons from g-C_3_N_4_ demonstrated the ability to reduce molecular oxygen into superoxide anions, further participating in the degradation of dyes. These generated active species (h^+^, ·O_2_^−^ and ·OH) eventually degraded TC dye pollutants into harmless substances such as CO_2_ and H_2_O.

Similarly, Nong et al. [115] developed a Z-Scheme NiCo-LDH/g-C_3_N_4_ heterojunction to achieve highly effective photodegradation of tetracycline hydrochloride (TC) and hydrogen production. This material significantly enhanced the photocatalytic performance by compounding NiCo-LDH nanosheets (LDH) with g-C_3_N_4_ (CN) to construct a tight heterostructure. A Z-Scheme photocatalysis mechanism was put forward for the nanocomposite material. Under visible light, LDH and CN were simultaneously stimulated, and electrons migrated from the valence bands to CB, while keeping holes on valence bands. Because the conduction band (−0.83 eV vs. NHE) of LDH possessed a more negative position relative to that of CN (−0.47 eV vs. NHE), hence electrons spontaneously transferred from CN’s CB to LDH’s VB and combined with the VB holes of LDH. These processes followed a Z-shaped mechanism, causing strong reducing electrons to accumulate on LDH’s CB and strong oxidizing holes to accumulate on CN’s VB. The accumulated electrons exhibited strong reducing capacity for oxygen reduction to ·O_2_^−^, while the holes of CN were able to oxidize H_2_O to ·OH. These active species (·O_2_^−^ and ·OH, h^+^) possessed robust oxidizing capabilities and were capable of degrading TC organic pollutants efficiently. This Z-shaped heterojunction design retained the material’s high redox capacity while restraining the electron-hole pairs direct recombination. By optimizing the charge carrier migration path and the generation of active species, it achieved efficient degradation of pollutants. The findings demonstrated that the optimized material enabled an 88.2% tetracycline removal under visible-light irradiation.

#### 4.1.3. S-Scheme Heterojunction

The S-Scheme heterojunction represents a recent refinement and precision in the mechanism of Z-Scheme heterojunctions [116]. This configuration differs fundamentally from conventional Z-Scheme systems through its strategic combination of a reduction photocatalyst (RP) featuring lower work function and elevated Fermi level with an oxidation photocatalyst (OP) possessing higher work function and reduced Fermi level, achieved through staggered stacking [117]. Three synergistic pathways—inherent electric field formation, energy band realignment, and electrostatic forces—collectively enable effective spatial segregation of electron-hole pairs within both RP and OP components, ultimately improving the material’s photocatalytic performance [118].

For example, Feng and colleagues [119] developed a 3D flower-like oxygen-vacancy-rich g-C_3_N_4_/NiZnAl-LDH S-Scheme heterojunction photocatalytic material via a hydrothermal method. The unique 3D flower-like structure offered an expanded surface area and multiple reactive centers, while the oxygen vacancies (OVs) further boosted photon absorption efficiency and charge carrier separation. The S-Scheme design successfully prevented the electron-hole pair recombination while preserving the high redox capability of the material, significantly improving the decomposition of methyl orange (MO) and tetracycline (TC). When exposed to visible light, g-C_3_N_4_ (RP) and NiZnAl-LDH (OP) were simultaneously excited, generating electrons and holes. The Fermi level disparity between RP and OP induces an interfacial electric field oriented from RP to OP, facilitating selective recombination of OP’s electrons with RP’s holes while preserving RP’s conduction band electrons for redox reactions. This selective recombination mechanism (S-Scheme) avoided the loss of redox capability observed in traditional Type-II heterojunction photocatalysts. The retained e^−^ within g-C_3_N_4_’s conduction band exhibited strong reduction ability, reducing O_2_ to ·O_2_^−^, whereas the valence band h^+^ of NiZnAl-LDH possessed strong oxidation ability, oxidizing OH^−^ or H_2_O to ·OH. Under attack by the aforementioned active species (h^+^, ·O_2_^−^, and ·OH), TC and MO were ultimately decomposed into harmless small-molecule products, even CO_2_ and H_2_O. This study resolved the key contradiction between carrier recombination and redox capability in photocatalysis through the co-design of S-Scheme heterostructure and oxygen vacancies. Compared to traditional Type-II or Z-Scheme heterojunctions, the S-Scheme heterojunction mechanism enabled efficient separation and directional migration of carriers via built-in electric field as well as band bending, while preserving the strong reduction ability of RP and strong oxidation ability of OP, effectively enhancing photocatalytic performance and providing a novel approach for highly effective photodegradation of organic pollutants [120].

### 4.2. Synergistic Adsorption-Photocatalysis Mechanism

Beyond enhancing photocatalytic activity through heterojunctions, a distinct merit of g-C_3_N_4_/LDH composites lies in their unique “adsorption-photocatalysis” synergy. The superior adsorption capacity of LDHs concentrates pollutant molecules from water onto the catalyst surface, significantly increasing their local concentration near photocatalytic active sites [121]. This enrichment markedly accelerates subsequent photodegradation rates, achieving efficient collaboration between adsorption and photocatalysis to improve removal efficiency for low-concentration aquatic pollutants [122].

For instance, Yu and colleagues [50] developed a porous g-C_3_N_4_/MgZnAl-calcined (M-CN/cLDH) photocatalyst via templating. This composite featured a 3D flower-like structure that formed via self-assembly of stacked hybrid nanosheets, exhibiting exceptional synergistic adsorption-photocatalysis for degrading tetracycline antibiotics (e.g., Oxytetracycline(OTC), Doxycycline (DXC), Chlortetracycline (CTC), TC) in high-salinity seawater. The calcined layered double hydroxide(cLDH)’s large specific surface area and porous nanostructure adsorbed antibiotics through electrostatic attraction, hydrogen-bond, and π-π conjugation, enriching them at reactive interfaces. Simultaneously, the tight heterojunction between cLDH and g-C_3_N_4_ facilitated electron-hole separation: electrons in g-C_3_N_4_’s CB reacted with oxygen to generate ·O_2_^−^, while holes in cLDH’s VB directly oxidized adsorbed antibiotics, aided by ·OH from ·O_2_^−^ conversion. Adsorption enriched pollutants near active sites, accelerating degradation, while photocatalysis decomposed pollutants and regenerates adsorption sites. This dynamic “adsorption-enrichment-degradation-desorption” cycle sustained a 95.73% OTC removal rate in high-salinity seawater and reduced toxicity of degradation products (confirmed by Liquid Chromatography-Mass Spectrometry, LC-MS), demonstrating high efficacy in complex water purification.

### 4.3. Persulfate-Assisted Photocatalytic Mechanism

To elevate the efficiency of degrading refractory organic pollutants, researchers have integrated photocatalysis with advanced oxidation processes (AOPs) by introducing persulfate (PS), such as peroxymonosulfate (PMS, HSO_5_^−^) or peroxydisulfate (PDS, S_2_O_8_^2−^), into photocatalytic systems, establishing a PS-assisted advanced oxidation system [123].

In the g-C_3_N_4_/LDHs photocatalytic system, PS activation primarily occurs via three pathways:

(1) Photoactivation: PS can be directly activated by light (especially UV), cleaving the O-O bond to produce sulfate radicals (·SO_4_^−^) [124]. ·SO_4_^−^ exhibits stronger oxidizing power (E^0^ = 2.5–3.1 V), longer lifespan, and broader pH applicability than ·OH [125]. In g-C_3_N_4_/LDHs, light simultaneously excites the semiconductor and PS, producing multiple reactive species (·OH, ·O_2_^−^, ·SO_4_^−^), forming a synergistic “combined approach” that significantly enhances degradation [126].

(2) Photogenerated electron activation: Visible light-excited g-C_3_N_4_ generates conduction-band electrons (e^−^) with strong reducing capability [127]. These electrons efficiently reduce PS, breaking its peroxy bond (-O-O-) to yield ·SO_4_^−^ [128]:

e^−^ + S_2_O_8_^2−^ → ·SO_4_^−^ + SO_4_^2−^

e^−^ + HSO_5_^−^ → ·SO_4_^−^ + OH^−^ (acidic) or ·SO_4_^−^ + ·OH (alkaline)

(3) Metal ion catalytic activation: If LDHs contain variable-valence metal ions (e.g., Co^2+^, Fe^2+^, Cu^2+^), these ions catalyze PS activation via Fenton-like reactions, generating ·SO_4_^−^ and ·OH (e.g., Co^2+^ + S_2_O_8_^2−^ → Co^3+^ + ·SO_4_^−^ + ·SO_4_^2−^) [129]. Photogenerated electrons reduce high-valence metal ions (e.g., Fe^3+^ → Fe^2+^), enabling metal ion regeneration and sustained PS activation, establishing a synergistic “photocatalytic–Fenton-like” cyclic system [130].

Zeng et al. [131] fabricated a CoAl-LDH/g-C_3_N_4_ (CoAl-LDH/CN) heterostructure via electrostatic self-assembly that used g-C_3_N_4_ nanosheets and exfoliated CoAl-LDH and employed a peroxymonosulfate (PMS)-assisted photocatalytic mechanism to efficiently degrade sulfadiazine (SDZ), while elucidating the catalytic process. The photoinduced electrons (e^−^) from CoAl-LDH’s conduction band transferred to g-C_3_N_4_’s CB, subsequently reacting with dissolved O_2_ to form ·O_2_^−^. Simultaneously, the e^−^ were capable of activating PMS to yield ·SO_4_^−^ and ·OH. Additionally, ·OH and h^+^ can combine with superoxide species to form highly reactive singlet oxygen (^1^O_2_). Moreover, XPS results indicated that Co^2+^ participates in activating PMS, generating sulfate radicals (·SO_4_^−^) through a series of reactions. In this mechanism, CoAl-LDH primarily facilitated PMS activation for radical generation, meanwhile g-C_3_N_4_ played a double role: acting as an acceptor to prolong charge separation, and directly participating in PMS activation. This synergistic mechanism enhanced the production efficiency of ROS, including ·OH, ·SO_4_^−^, and ^1^O_2_, significantly improving pollutant degradation performance, achieving a rapid degradation of SDZ with a removal efficiency of 87.1% within 15 min.

Shen and colleagues [132] developed a new composite through anchoring CoFe layered double oxide on g-C_3_N_4_ (CoFe-LDO/g-C_3_N_4_) for efficient PMS activation to degrade paracetamol in water. The material was fabricated using a straightforward co-precipitation method combined with calcination, activating PMS through a dual mechanism involving free radicals and electron transfer, which achieved rapid pollutant degradation. Herein, Co^2+^ and Fe^2+^ in CoFe-LDO were key sites for PMS activation. PMS (HSO_5_^−^) decomposed under activation by Co^2+^ and Fe^2+^ to generate ·SO_4_^−^ and ·OH (Co^2+^/Fe^2+^ + HSO_5_^−^ → Co^3+^/Fe^3+^ + ·SO_4_^−^ + OH^−^). Subsequent regeneration of active metal ions occurred through PMS-mediated reduction (Co^3+^/Fe^3+^ + HSO_5_^−^ → Co^2+^/Fe^2+^ + ·SO_5_^−^ + H^+^). During interaction with aqueous media, ·SO_4_^−^ can also produce ·OH (·SO_4_^−^ + H_2_O → SO_4_^2−^ + ·OH + H^+^, ·SO_4_^−^ + OH^−^ → SO_4_^2−^ + ·OH). Furthermore, self-decomposition of PMS can yield ·O_2_^−^ (HSO_5_^−^ → SO_5_^2−^ + H^+^, SO_5_^2−^ + HSO_5_^−^ → SO_5_^2−^ + HSO_4_^−^+ O_2_), and the resulting active species (·SO_4_^−^, ·OH, and ·O_2_^−^) possessed strong oxidizing capabilities, enabling rapid attack and degradation of pollutant molecules. Concurrently, g-C_3_N_4_ served as an electron-donating species, lowering the oxidation states of Co and Fe and promoting electron migration between contaminant molecules toward PMS. Specifically, pollutant molecules were oxidized on the catalyst surface, releasing electrons. These electrons were transferred through g-C_3_N_4_ to CoFe-LDO, reducing Co^3+^/Fe^3+^ to Co^2+^/Fe^2+^, while PMS accepted electrons and was activated, further generating ROS such as SO_4_^−^ and ·OH, thereby sustaining the catalytic cycle.

## 5. Specific Application Cases in Water Pollution Control

g-C_3_N_4_/LDHs composites demonstrate significant application potential in treating various water pollutants due to their unique synergistic advantages. In Table 2, examples of the latest research work of g-C_3_N_4_/LDHs composite materials in water pollution treatment are presented. This section categorizes recent research progress on their degradation of organic dyes, drugs, and phenolic compounds.

### 5.1. Organic Dyes

Organic dyes, characterized by high chromaticity, strong toxicity, and poor biodegradability, are primary pollutants in industrial effluents from dyeing and printing sectors. The degradation of dyes by g-C_3_N_4_/LDHs composites represents one of the most extensively studied and effective applications [137]. This is largely attributed to the electrostatic adsorption between the positively charged layers of LDHs and organic dyes (mostly anionic), which forms an “adsorption-enrichment-degradation” synergistic effect, significantly increasing the local pollutant concentration and shortening the reaction distance between reactive species (·O_2_^−^, ·OH) and pollutants.

**Figure 13:**
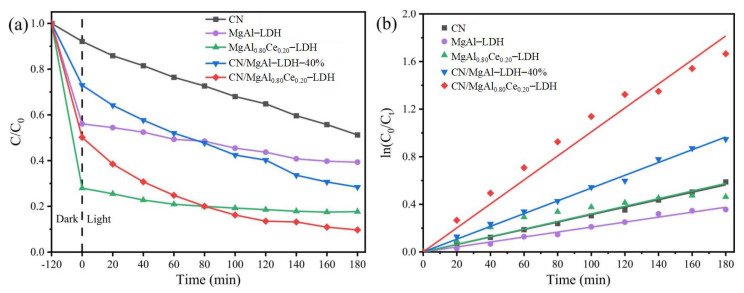
(**a**) CR adsorption-photocatalysis activity, (**b**) pseudo-first-order kinetic curves for CR photocatalytic degradation of CN, MgAl-LDH, MgAl_0.80_Ce_0.20_-LDH, CN/MgAl-LDH-40% and CN/MgAl_0.80_Ce_0.20_-LDH. Reprinted with permission from Ref. [138].

For example, Huang and colleagues [138] synthesized a layered composite photocatalyst, CN/MgAl_0.80_Ce_0.20_-LDH, through a one-step solvothermal technology by combining g-C_3_N_4_(CN) with cerium modified MgAl-LDH. Studies revealed that cerium doping and the CN-LDH synergy significantly enhanced the composite’s surface characteristics and photogenerated charge separation capability, enabling an adsorption-photocatalytic degradation mechanism. During degradation, CN/MgAl_0.80_Ce_0.20_-LDH first adsorbed dye molecules through its elevated surface area and rich active sites. Experiments showed near 49% adsorption rate for 50 mg/L Congo Red (CR) under dark conditions (Figure 13a). Subsequently, under LED visible light (400–760 nm), the material’s photocatalytic activity was activated, achieving over 90% CR degradation within 180 min—far exceeding the 48.77% efficiency of pure CN. Figure 13b further confirmed its superiority via pseudo-first-order kinetic fitting.

Jie et al. [122] pioneered the synthesis of three innovative ZnCo-LDHs/g-C_3_N_4_ composites via triethanolamine-assisted layered construction technology for the efficient degradation of Sunset Yellow (SY) dye. The composites combined the high adsorptive capability of ZnCo-LDHs with g-C_3_N_4_’s photocatalytic activity, achieving synergistic enhancement. Experiments confirmed the generation of ·O_2_^−^, ·OH, and ^1^O_2_ during photocatalysis, collectively participating in SY oxidation. Of the three synthesized samples, ZnCo-LDHs/g-C_3_N_4_-3 exhibited optimal performance, degrading 99.6% of 75 mg/L SY within 90 min and retaining 84.3% activity after three cycles, highlighting structural stability and reusability for efficient dye wastewater treatment.

### 5.2. Drugs

Drugs especially antibiotics have emerged as a class of trace organic pollutants of significant concern in recent years. Due to their stable molecular structures, antibiotics are recalcitrant to conventional removal methods [139]. The performance bottleneck in drugs degradation can be effectively broken through by elemental doping (e.g., F, Cl co-doping) to narrow the band gap and constructing heterojunctions [98].

For example, Wei and colleagues [133] efficiently degraded tetracycline hydrochloride (TCH) by constructing an S-Scheme heterojunction. They combined MgFeTi-LDH with g-C_3_N_4_ through a self-assembly process, narrowing the material’s band gap from 2.78 eV to 2.67 eV, improving visible spectrum utilization and enhancing light energy utilization. The establishment of an S-Scheme heterojunction facilitated superior separation of photoinduced electron-hole pairs, suppressing recombination and consequently boosting catalytic activity. Additionally, the cooperative interaction between the Fe^3+^/Fe^2+^ redox cycle and sodium persulfate(SPS) activation generated abundant reactive radicals (e.g., ·OH, ·SO_4_^−^, and ·O_2_^−^), which exhibited strong oxidative capacity for efficient TCH degradation. Experimental results demonstrated near-complete TCH removal (approximately 100%) under visible light, with negligible performance degradation following four cycles of use, indicating outstanding stability and promising potential for industrial application. This study provided an economical, efficient, and sustainable solution for mitigating antibiotic contamination in water bodies, showing promising prospects for practical wastewater treatment.

**Figure 14:**
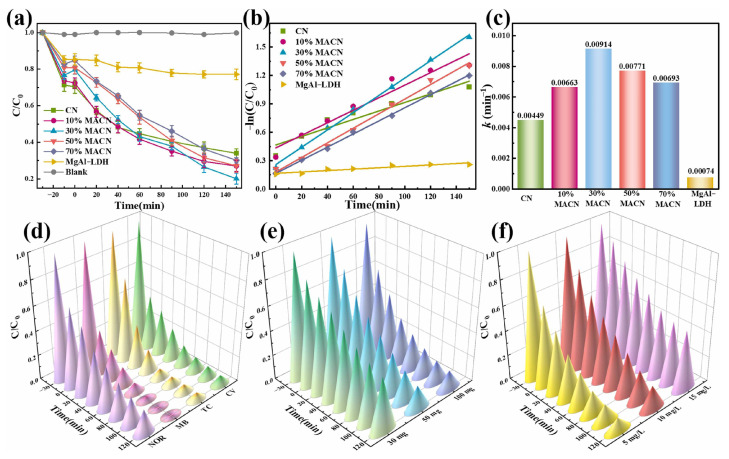
(**a**) CIP degradation graphs, (**b**) kinetics of the first order, (**c**) rate constants of degradation reactions, (**d**) photocatalytic degradation profiles, (**e**) 30%MACN dosage effect on CIP degradation, (**f**) initial CIP concentration impact on degradation. Reprinted with permission from Ref. [134].

Similarly, Qin et al. [134] synthesized a MgAl-LDH/g-C_3_N_4_ nanocomposite through self-assembly technology for efficient photocatalytic degradation of ciprofloxacin (CIP) in water. Uniform anchoring of MgAl-LDH nanosheets onto g-C_3_N_4_ nanosheets constructed a compact heterojunction, significantly enhancing charge carrier separation, thus achieving superior photocatalytic performance. As shown in Figure 14a, with the optimized 30% MACN, CIP degradation reached 80.1% within 150 min of visible-light illumination, and its first-order rate constant (0.00914 min^−1^) was 2.04-fold greater than pristine g-C_3_N_4_ (Figure 14b,c). Figure 14e,f examined the influence of dosage and CIP concentration. The composite also showed strong stability and recyclability, retaining more than 70% efficiency of degradation subsequent to four usage cycles, and demonstrated excellent degradation performance for other organic pollutants such as norfloxacin, methylene blue, tetracycline, and crystal violet (Figure 14d).

### 5.3. Phenolic Compounds

Phenols and their derivatives (e.g., phenol, bisphenol A) are common toxic organic contaminants in wastewater originating from industries including chemical manufacturing, coking, and pesticides [140]. They exhibit carcinogenic, teratogenic, and mutagenic properties, and their stable benzene ring structure renders them recognized as refractory organic pollutants [141]. The degradation rate of phenolic compounds is usually lower than that of organic dyes and antibiotics [135,136]. The mineralization efficiency of phenolic compounds can be enhanced by adopting novel preparation methods or constructing multi-component heterojunctions to broaden the spectral response range.

For instance, Li et al. [81] prepared a visible-light-responsive Z-Scheme heterojunction photocatalyst (ZnAl-LDH/g-C_3_N_4_) through polydopamine (PDA) cross-linking. ZnAl-LDH initially synthesized via co-precipitation, underwent thermal treatment at 300–600 °C for two hours to form ZnAl-LDO (mixed metal oxides). The calcined ZnAl-LDO was mixed with g-C_3_N_4_ dispersion, dopamine hydrochloride, and buffer, followed by 24 h stirring at 80 °C. PDA bridged ZnAl-LDO nanosheets and g-C_3_N_4_, with electrostatic/π-π interactions anchoring them into a “sheet-sheet” contact heterojunction. This method avoided complex high-temperature/pressure operations (e.g., hydrothermal method), optimized interfacial contact via PDA modulation, and enhanced charge transfer and photocatalytic performance, achieving 99.04% p-nitrophenol degradation under visible light (>420 nm).

Li et al. [142] synthesized a novel porous Ag_3_PO_4_/(Cs, Rb)_x_WO_3_/g-C_3_N_4_/CoAl-LDH photocatalyst and applied it to the highly effective photodegradation of phenol, along with its derivatives including 2-chlorophenol (2-CP) and 2-nitrophenol (2-NP). Ag_3_PO_4_ demonstrated high quantum efficiency and superior photocatalytic activity; (Cs, Rb)_x_WO_3_ exhibited broad-spectrum light absorption across UV, visible, and near-infrared regions, with mixed valence states (W^6+^ and W^5+^) and oxygen vacancies facilitating the migration of photogenerated electrons. The layered structure as well as π-conjugated system of g-C_3_N_4_ boosted charge separation efficiency, while CoAl-LDH provided favorable redox potential and photochemical absorption properties. The cooperative actions of these components and the porous structural design observably improved the composite’s photocatalytic activity. Experimental findings revealed that visible-light-driven degradation rates of phenol, 2-CP, and 2-NP reached 94.6%, 97.5%, and 98.5%, respectively; moreover, the efficiency still exceeded 80% following five recycling cycles, which attests to the material’s outstanding stability and reusability.

### 5.4. Performance Comparison and Challenge Analysis

Table 3 compares the performance of some g-C_3_N_4_/LDHs composite materials in the removal of various pollutants and hydrogen production through photocatalysis. Overall, pollutant degradation relies on the “adsorption–enrichment–degradation” synergistic mechanism, which exhibits strong compatibility with various synthesis methods—most techniques such as mechanical mixing, self-assembly, and hydrothermal methods can achieve efficient degradation [17,48,65,72,100,136]. Its performance is closely related to the structural stability of pollutants. Due to the stable structure of the phenolic benzene ring, its degradation rate is generally lower than that of dyes and drugs. In contrast, hydrogen production performance primarily depends on photogenerated electrons with high reduction potential and efficient charge separation, which are highly reliant on precise structural design and synthesis processes. The hydrothermal method, capable of constructing intimate heterojunction interfaces and effectively retaining electrons with high reduction potential, typically demonstrates higher activity in photocatalytic hydrogen evolution, while simple processes struggle to achieve high hydrogen production rates [69,70,73,97].

It is worth noting that, compared with the biochar-based photocatalysts developed in recent years (such as Biochar/Fe-TiO_2_), g-C_3_N_4_/LDHs has more advantages in structural controllability and mechanism diversity, but still faces greater challenges in cost control and large-scale preparation [143]. For example, the layer composition and interlayer ions of LDHs can be adjusted, facilitating the construction of various heterojunctions such as Type-II and Z/S types, while biochar mainly serves as an electron acceptor and adsorption carrier. Under visible light, both can achieve degradation efficiency of over 80% for dyes, but g-C_3_N_4_/LDHs shows broader adaptability in the simultaneous removal of complex pollutants and photocatalytic hydrogen production. Although g-C_3_N_4_/LDHs exhibits excellent performance at the laboratory scale, its transition to practical application still faces multiple challenges: the scalability of the synthesis method, cycling stability, raw material cost (including the high cost of preparing LDHs such as Co^2+^ and Ni^2+^), and the adaptability to complex real water bodies. Future research should pay more attention to the long-term performance of the material in real wastewater and the engineering amplification path.

## 6. Conclusions and Prospects

In summary, g-C_3_N_4_/LDHs composites had become a highly promising type of photocatalysts applied in water remediation, leveraging the synergetic combination of g-C_3_N_4_’s photoactivity and LDHs’ superior adsorption and structural tunability. Through various synthesis strategies, these composites formed effective heterojunctions (notably Z-Scheme and S-Scheme) that significantly enhanced charge separation, broadened the visible-light response, and integrated adsorption with photocatalysis. This results in markedly improved performance for degrading diverse pollutants, removing heavy metals, and disinfecting water. However, the transition from laboratory promise to practical application is hindered by challenges including the need for a deeper mechanistic understanding of interfacial charge dynamics, the development of synthesis methodologies featuring scalability and cost efficiency, and the demonstration of long-term stability and efficacy in complex, real-water environments.

Future endeavors should pivot towards precision design guided by computational screening and machine learning to tailor material properties at the atomic level. Concurrently, we should focus on advancing scalable manufacturing and immobilization techniques to facilitate practical deployment. Expanding the functionality of these systems by integrating them with other processes like photothermal conversion, membrane filtration, or electrocatalysis, which present a compelling path for tackling complex pollution scenarios and broadening applications to CO_2_ reduction and nitrogen fixation. Ultimately, the successful translation of this technology hinges on application-oriented engineering, requiring the design of efficient photoreactors and rigorous pilot-scale studies to validate its techno-economic feasibility for sustainable water treatment.

## Figures and Tables

**Table 1:** Comparison of mainstream preparation methods for g-C_3_N_4_/LDHs composites.

Preparation Method	Advantages	Limitations	Scalability	Cost	Industrial Application Potential
Electrostatic Self-Assembly	Mild conditions, preserved component structure, tight 2D/2D heterojunctions	Weak interfacial force compared to hydrothermal	High	Low	High (simple equipment, low energy consumption)
Co-Precipitation	Uniform in situ growth, tight interfacial contact, low cost	Strict pH control, limited morphology tunability	Medium	Low	Medium (mature process, but pH regulation needs optimization)
Hydrothermal/ Solvothermal	High crystallinity, robust interfacial synergy	High temperature/pressure, complex equipment	Low	High	Low (high energy consumption, batch production only)
Physical/ Mechanical Mixing	Operational simplicity, low cost, time-saving	Weak interfacial interaction, uneven dispersion	Very High	Very Low	High (suitable for bulk production)
Calcination	Enhanced stability, modified band structure	Risk of component oxidation/decomposition	Medium	Medium	Medium (needs precise temperature control)
Other novel methods	Tailored interfaces, unique morphologies, possible selectivity	Often complex, less established, may require special equipment	Variable	Variable	Low (Niche high-performance applications)

**Table 2:** The latest research work of g-C_3_N_4_/LDHs composite in water pollution treatment.

Photocatalyst	Preparation Technique	Analyte	Maximum Degradation Rate (%)	Reference
g-C_3_N_4_/@CoAl-LDH	Hydrothermal Method	Crystal violet (CV)	99% (60 min)	[14]
g-C_3_N_4_@NiFe-LDH	Hydrothermal Method	Rhodamine B (RhB)	99% (240 min)	[16]
ZnAl-LDH/g-C_3_N_4_	Calcination Method	Phenol	90.25% (270 min)	[17]
CoNiFe-LDH/g-C_3_N_4_	Hydrothermal Method	Methylene blue (MB)	98.29% (35 min)	[34]
MgAl-LDH@ g-C_3_N_4_@ Ag_3_PO_4_	Hydrothermal Technology	Methylene blue (MB)	99% (45 min)	[48]
CoFe-LDH/g-C_3_N_4_	Coprecipitation Method	Tetracycline (TC)	83.8% (180 min)	[49]
MgAl-LDH/g-C_3_N_4_	Self-Assembly Method	Methyl orange (MO)	93.58% (120 min)	[52]
S, P co-doped g-C_3_N_4_/ZnCr-LDH	Coprecipitation Method	Ciprofloxacin (CIP)	95% (90 min)	[65]
ZnTi-LDH/g-C_3_N_4_	Mechanical Mixing Method	Methyl orange (MO)	100% (90 min)	[72]
Zn-Al LDH/g-C_3_N_4_	Microwave Irradiation Technology	Ciprofloxacin (CIP)	84.1%(150 min)	[79]
yP-LDH_TCN	Polydopamine Cross-Linking Method	P-nitrophenol (4-NP)	99.04% (120 min)	[81]
Ag-doped g-C_3_N_4_@NiFe-LDH	Spin Disk Reactor	Rhodamine B (RhB)	99% (240 min)	[82]
CuFe-LDH/g-C_3_N_4_	Hydrothermal Technology	Methylene blue (MB)	97% (120 min)	[91]
g-C_3_N_4_/LDH-OVs	Mechanical Grinding method	Tetracycline (TC)	95%(60 min)	[92]
Vn-CN/Co/LDH	Hydrothermal Technology	Ofloxacin (OFX)	100%(15 min)	[93]
FCCN/LDH	Coprecipitation Method	Tetracycline (TC)	95.5% (120 min)	[98]
NiAl-LDH/Ag/g-C_3_N_4_	Hydrothermal Method	Methylene blue (MB)	99.60% (160 min)	[99]
Au/LDH/CN	Self-Assembly Technology	Tetracycline (TC)	97.14%(180 min)	[100]
g-C_3_N_4_/NiAl-LDH/CeO_2_	Hydrothermal Method	Rhodamine B (RhB)	98% (350 min)	[101]
CuAl-LDH/g-C_3_N_4_	Hydrothermal Method	Cresol Red	97% (90 min)	[102]
CN/MgAl_0.80_Ce_0.20_-LDH	Hydrothermal Technology	Congo red (CR)	90.33% (180 min)	[104]
CoAl-LDH@g-C_3_N_4_	Impregnation Technology	Brilliant black dye	79% (240 min)	[108]
NiAl-LDH/g-C_3_N_4_	Hydrothermal Technology	Tetracycline (TC)	98.5% (60 min)	[113]
ZnAlS_x_@ g-C_3_N_4_	Hydrothermal Technology	Tetracycline (TC)	94.05% (180 min)	[114]
NiCo-LDH/g-C_3_N_4_	Physical Mixing Method	Tetracycline (TC)	88.2% (70 min)	[115]
g-C_3_N_4_/NiAl-LDH/CeO_2_	Hydrothermal Method	Rhodamine B (RhB)	98% (350 min)	[122]
CoAl-LDH/g-C_3_N_4_	Self-Assembly Method	Sulfadiazine (SDZ)	87.1% (15 min)	[131]
MgFeTi-LDH/g-C_3_N_4_	Self-Assembly Method	Tetracycline (TC)	100% (60 min)	[133]
MgAl-LDH/g-C_3_N_4_	Self-Assembly Method	Ciprofloxacin (CIP)	80.1% (150 min)	[134]
g-C_3_N_4_/ZnAl-LDH	Calcination Method	Phenol	79.35% (300 min)	[135]
ZnTi-LDH/g-C_3_N_4_/Ag	Self-Assembly Method	Phenol	76.6% (180 min)	[136]

**Table 3:** Performance comparison of typical research on g-C_3_N_4_/LDHs composite materials.

Photocatalyst	Preparation Technique	Analyte	Maximum Degradation Rate (%)	Key Advantages/Features	Reference
ZnTi-LDH/g-C_3_N_4_	Mechanical mixing method	Methyl orange (MO)	100% (90 min)	Tight sheet-on-sheet interfacial contact, simple preparation process	[72]
MgAl-LDH@ g-C_3_N_4_@ Ag_3_PO_4_	Hydrothermal Method	Methylene blue (MB)	99% (45 min)	Tight interface, enhanced adsorption, wide visible light response range	[48]
S, P co-doped g-C_3_N_4_/ZnCr-LDH	Coprecipitation Method	Ciprofloxacin (CIP)	95% (90 min)	Intimate 2D/2D contact, abundant defect sites	[65]
Au/LDH/CN	Self-Assembly Method	Tetracycline (TC)	97.14%(180 min)	Ternary heterojunction synergy system, efficient interface charge separation	[100]
ZnAl-LDH/g-C_3_N_4_	Calcination Method	Phenol	90.25% (270 min)	Narrowed bandgap, good visible-light response	[17]
ZnTi-LDH/g-C_3_N_4_/Ag	Self-Assembly Method	Phenol	76.6% (180 min)	Simple assembly process, SPR effect	[136]
xNiCoAlCN	Physical Mixing Method	Photocatalytic H_2_ evolution	338 μmol·h^−1^·g^−1^	Simple, low-cost, forming close interface, S-Scheme charge transfer	[73]
NiCo-LDH/g-C_3_N_4_	Hydrothermal Method	Photocatalytic H_2_ evolution	3125 μmol·h^−1^·g^−1^	Z-Scheme heterojunction, strong reduction capability	[69]
g-C_3_N_4_/CoAl-LDH	Hydrothermal Method	Photocatalytic H_2_ evolution	3677.5 μmol·h^−1^·g^−1^	Mesoporous structure, heterojunction, interfacial hydrogen bonds	[70]
Mxene/S-g-C_3_N_4_/NiAl-LDH	Hydrothermal Method	Photocatalytic H_2_ evolution	72630 μmol·h^−1^·g^−1^	S-Scheme, excellent hierarchical structure, tight interface	[97]

## Data Availability

No new data were created or analyzed in this study.

## References

[1] Han M., Liu Z., Huang S., Zhang H., Yang H., Liu Y., Zhang K., Zeng Y. (2024). Application of Biochar-Based Materials for Effective Pollutant Removal in Wastewater Treatment. Nanomaterials.

[2] Qiu M., Hu B., Chen Z., Yang H., Zhuang L., Wang X. (2021). Challenges of organic pollutant photocatalysis by biochar-based catalysts. Biochar.

[3] Tamyiz M. (2025). Ecofriendly synthesis and characterization of oxygen-enriched g-C_3_N_4_ from diverse precursors for efficient organic dye decontamination. Turk. J. Chem..

[4] Hubab M., Gilani I.E., Al-Ghouti M.A. (2025). Metal-organic frameworks: A promising solution for addressing phenol pollution and promoting environmental sustainability. Environ. Technol. Innov..

[5] Siwińska-Ciesielczyk K., Andrzejczak A., Paukszta D., Piasecki A., Moszyński D., Zgoła-Grześkowiak A., Jesionowski T. (2021). Synthesis of selected mixed oxide materials with tailored photocatalytic activity in the degradation of tetracycline. Materials.

[6] Sumithra B., Saravanan V., Ramalingan C., Sivaganesh D., Lakshmanan P., Geetha D. (2025). Harnessing sunlight: Unlocking superior photocatalytic activity of g-C_3_N_4_/MnWO_4_ heterojunction photocatalysts for degradation of hazardous compounds. Tungsten.

[7] Chen J., Lu J., Lang R., Wang C., Bao S., Li Y., Fan M. (2025). Enhanced and selective photocatalytic reduction of CO_2_ to CH_4_ using a Pt-loaded CuPc/g-C_3_N_4_ Z-Scheme heterojunction catalyst. Green Energy Environ..

[8] Wang H., Wang T., Zhu Z., Ren S., Huang Y., Qian S., Tang W., Yin X., Niu H., Wang X. (2025). Construction of Multifunctional Photothermal/Photocatalytic Materials Based on the Principle of Three Primary Colors: A Case Study of g-C_3_N_4_/Ag_2_CrO_4_. Carbon Energy.

[9] Bao T., Li X., Li S., Rao H., Men X., She P., Qin J. (2025). Recent advances of graphitic carbon nitride (g-C_3_N_4_) based materials for photocatalytic applications: A review. Nano Mater. Sci..

[10] Yang R., Mu W., He L., Meng J., Bi X., Luo W., Luo S., Lei X. (2025). CdS/NiCoAl-LDH heterojunction for superior photocatalytic hydrogen production and stability in water splitting. Chem. Eng. J..

[11] Gao Q., Wang M., Zhu Y., Chai Y., Liu B. (2025). A band structure modulated 1D/2D CdS/MgAl-LDH S-Scheme heterojunction toward simultaneous photocatalytic removal of tetracycline and hexavalent chromium. Appl. Surf. Sci..

[12] Lu Y., Wang X., Xu X., Wu P., Li Z., Zhang G. (2025). Hierarchically structured CoAl-LDH derived hybrid membrane for synergistic solar-driven water purification and photocatalytic CO_2_ reduction. Desalination.

[13] Li C., Ding G., Wang P., Liu K., Yang B., Liao G. (2025). Emerging frontiers of nickel–aluminium layered double hydroxide heterojunctions for photocatalysis. Dalton Trans..

[14] Madhan D., Devabharathi V., Muthusamy S., Subramani T. (2025). CoAl-LDH incorporated g-C_3_N_4_ nanosheets: A dual-function photocatalyst for hydrogen production and dye degradation. Ionics.

[15] Song B., Zeng Z., Zeng G., Gong J., Xiao R., Ye S., Chen M., Lai C., Xu P., Tang X. (2019). Powerful combination of g-C_3_N_4_ and LDHs for enhanced photocatalytic performance: A review of strategy, synthesis, and applications. Adv. Colloid Interface Sci..

[16] He Y., Zhou S., Wang Y., Jiang G., Jiao F. (2021). Fabrication of g-C_3_N_4_@ NiFe-LDH heterostructured nanocomposites for highly efficient photocatalytic removal of rhodamine B. J. Mater. Sci. Mater. Electron..

[17] Tripathi A., Hussain C.M. (2021). ZnAl-LDH and B-impregnated polymeric semiconductor (g-C_3_N_4_) for solar light-driven photocatalysis to treat phenolic effluent. Sustain. Mater. Technol..

[18] Bai Y., Dong W., Zhang Q., She H., Chen X., Wang Q. (2025). Preparation of FeCu-LDH/g-C_3_N_4_ catalysts and synergistic activation of potassium persulfate for photocatalytic significant degradation of tetracycline. J. Alloys Compd..

[19] Zhong T., Huang W., Yao Z., Long X., Qu W., Zhao H., Tian S., Shu D., He C. (2024). Engineering of Graphitic Carbon Nitride (g-C_3_N_4_) Based Photocatalysts for Atmospheric Protection: Modification Strategies, Recent Progress, and Application Challenges. Small.

[20] Ong W.J., Tan L.L., Ng Y.H., Yong S.T., Chai S.P. (2016). Graphitic carbon nitride (g-C_3_N_4_)-based photocatalysts for artificial photosynthesis and environmental remediation: Are we a step closer to achieving sustainability?. Chem. Rev..

[21] Ivanova V., Pimpilova M., Stoyanova M., Dimcheva N. (2025). Electrochemical Method for the Assay of Organic Peroxides Directly in Acetonitrile. Molecules.

[22] Zhang J., Wang L., Tang H., Li X., Jiang A., Wu Y., Xian S., Xia R., Li Y., Jiang Z. (2025). A highly luminescent 3-phenylfluoranthene-modified g-C_3_N_4_ derivative used as a metal-free phosphor in white light-emitting diodes. RSC Adv..

[23] Glažar D., Jerman I., Tomšič B., Chouhan R.S., Simončič B. (2023). Emerging and Promising Multifunctional Nanomaterial for Textile Application Based on Graphitic Carbon Nitride Heterostructure Nanocomposites. Nanomaterials.

[24] Zhu W., Yue Y., Wang H., Zhang B., Hou R., Xiao J., Huang X., Ishag A., Sun Y. (2023). Recent advances on energy and environmental application of graphitic carbon nitride (g-C_3_N_4_)-based photocatalysts: A review. J. Environ. Chem. Eng..

[25] Hayat A., Sohail M., Anwar U., Taha T.A., Qazi H.I.A., Amina, Ajmal Z., Al-Sehemi A.G., Algarni H., Al-Ghamdi A.A. (2023). A targeted review of current progress, challenges and future perspective of g-C_3_N_4_ based hybrid photocatalyst toward multidimensional applications. Chem. Rec..

[26] Gao M., Zhao M., Yang Q., Bao L., Chen L., Liu W., Feng J. (2025). A Review on Pre-, In-Process, and Post-Synthetic Strategies to Break the Surface Area Barrier in g-C_3_N_4_ for Energy Conversion and Environmental Remediation. Nanomaterials.

[27] Jing S., Xu Q., Wang H., Kannan P., Liang H., Brouzgou A., Wang R., Tsiakaras P. (2025). Improved photocatalytic production of hydrogen peroxide over graphitic carbon nitride doped with potassium salts. J. Colloid Interface Sci..

[28] Chen S., Zhang X., Li D., Wang X., Hu B., Guo F., Hao L., Liu B. (2024). Strategies for improving photocatalytic performance of g-C_3_N_4_ by modulating charge separation and current research status. Heliyon.

[29] Pausch I., Lohse H.-H., Schürmann K., Allmann R. (1986). Syntheses of Disordered and Al-Rich Hydrotalcite-Like Compounds. Clays Clay Miner..

[30] Jaramillo-Hernández C., Oestreicher V., Mizrahi M., Abellán Sáez G. (2023). Upscaling the urea method synthesis of CoAl layered double hydroxides. Beilstein J. Nanotechnol..

[31] Zhu Y., Wang J., Ma J. (2023). Recent Progress on Non-Carbon-Supported Single-Atom Catalysts for Electrochemical Conversion of Green Energy. Small Sci..

[32] Lu X., Xue H., Gong H., Bai M., Tang D., Ma R., Sasaki T. (2020). 2D layered double hydroxide nanosheets and their derivatives toward efficient oxygen evolution reaction. Nano-Micro Lett..

[33] Berede H.T., Andoshe D.M., Gultom N.S., Kuo D.H., Chen X., Abdullah H., Wondimu T.H., Wu Yn W.Y., Zelekew O.A. (2024). Photocatalytic activity of the biogenic mediated green synthesized CuO nanoparticles confined into MgAl LDH matrix. Sci. Rep..

[34] Zhu B., Xu Q., Bao X., Lu D., Yin H., Qin Y., Shen X.C. (2021). g-C_3_N_4_/CoNiFe-LDH Z-Scheme heterojunction for efficient CO_2_ photoreduction and MB dye photodegradation. Catal. Sci. Technol..

[35] Biswal L., Sahoo D.P., Mohanty U.A., Parida K. (2025). MXene Derived Ti_3_C_2_-TiO_2_ Coupled NiCo LDH: A 2D/3D Interfacial Engineered S-Scheme Heterojunction for Enhanced Photocatalytic H_2_O_2_ and H_2_ Production. Langmuir.

[36] Liu X., Zhou Y., Sun S., Bao S. (2023). Study on the behavior and mechanism of NiFe-LDHs used for the degradation of tetracycline in the photo-Fenton process. RSC Adv..

[37] Sarangi P.P., Das K.K., Sahu J., Mohanty U.A., Sahoo D.P., Parida K. (2025). Structurally Modulated NiV-LDH with CdMoSe-Quantum Dots: Unlocking the Active Centers at S-Scheme Heterojunctions for Stimulating Photocatalytic H_2_O_2_ Production and H_2_ Evolution. Inorg. Chem..

[38] Ghafuri H., Ghanbari N. (2024). Design and synthesis of LDH nano composite functionalized with trimesic acid and its environmental application in adsorbing organic dyes indigo carmine and methylene blue. Heliyon.

[39] Qu J., Sha L., Wu C., Zhang Q. (2019). Applications of mechanochemically prepared layered double hydroxides as adsorbents and catalysts: A mini-review. Nanomaterials.

[40] Fu Y., Fu X., Song W., Li Y., Li X., Yan L. (2023). Recent progress of layered double hydroxide-based materials in wastewater treatment. Materials.

[41] Qi H., Wolfe J., Fichou D., Chen Z. (2016). Cu_2_O photocathode for low bias photoelectrochemical water splitting enabled by NiFe-layered double hydroxide Co-catalyst. Sci. Rep..

[42] Mureseanu M., Cioatera N., Carja G. (2023). Fe-Ce/layered double hydroxide Heterostructures and their derived oxides: Electrochemical characterization and light-driven catalysis for the degradation of phenol from water. Nanomaterials.

[43] Yang Z., Xiong X., Yan X., Luo S., Zhang Y., Briseghella B., Marano G.C. (2023). NO_x_ degradation ability of Sg-C_3_N_4_/MgAl-CLDH nanocomposite and its potential application in cement-based materials. RSC Adv..

[44] Xia Y., Liang R., Yang M.Q., Zhu S., Yan G. (2021). Construction of chemically bonded interface of organic/inorganic g-C_3_N_4_/LDH heterojunction for Z-schematic photocatalytic H_2_ generation. Nanomaterials.

[45] Jiang R., Yu F., Duan X., Zhang J., Ren J., Yu X., Liu L., Feng C., Li C., Hu K. (2025). Study on heterojunctions-based g-C_3_N_4_/ZnCdS/MoO_3_ composite photocatalyst with a dual S-Scheme carrier transfer mechanism. J. Alloys Compd..

[46] Fui H., Gao S., Ma X., Huang Y. (2023). Facile fabrication of CoAl-LDH nanosheets for efficient rhodamine B degradation via peroxymonosulfate activation. RSC Adv..

[47] Patial S.K., Rani D., Garg M., Meena V.K., Pahuja M., Ghosh K., Singh S. (2025). Sustainable Electrochemical Synthesis of Porous g-C_3_N_4_ Nanosheets via 3D-Printed Platinized Electrodes for Enhanced Photocatalytic Activity. Langmuir.

[48] Salehi G., Bagherzadeh M., Abazari R., Hajilo M., Taherinia D. (2024). Visible light-driven photocatalytic degradation of methylene blue dye using a highly efficient Mg-Al LDH@ g-C_3_N_4_@ Ag_3_PO_4_ nanocomposite. ACS Omega.

[49] Li M., Chen M., Lee S.L.J., Lin S. (2023). Facile fabrication of a 2D/2D CoFe-LDH/g-C_3_N_4_ nanocomposite with enhanced photocatalytic tetracycline degradation. Environ. Sci. Pollut. Res..

[50] Yu Y., Chen D., Xu W., Fang J., Sun J., Liu Z., Chen Y., Liang Y., Fang Z. (2021). Synergistic adsorption-photocatalytic degradation of different antibiotics in seawater by a porous g-C_3_N_4_/calcined-LDH and its application in synthetic mariculture wastewater. J. Hazard. Mater..

[51] Zhang L., Li L., Sun X., Liu P., Yang D., Zhao X. (2016). ZnO-layered double hydroxide@ graphitic carbon nitride composite for consecutive adsorption and photodegradation of dyes under UV and visible lights. Materials.

[52] Hao T., Xu H., Sun S., Yu H., Qin Q., Song B., Li M., Shao G., Fan B., Wang H. (2024). Assembling flower-like MgAl-LDH nanospheres and g-C_3_N_4_ nanosheets for high efficiency removal of methyl orange. Ceram. Int..

[53] Zhu Z., Wang L., Zhang W., Hou C., Wang C., Zhao J. (2024). Ultrathin CuNi_2_Al-LDH nanosheets with enhanced electron transfer for visible-light-driven photo-fenton-like water decontamination. Chem. Eng. J..

[54] Ghosh D., Periyasamy G., Pandey B., Pati S.K. (2014). Computational studies on magnetism and the optical properties of transition metal embedded graphitic carbon nitride sheets. J. Mater. Chem. C.

[55] Ou B., Wang J., Wu Y., Zhao S., Wang Z. (2020). Efficient removal of Cr (VI) by magnetic and recyclable calcined CoFe-LDH/g-C_3_N_4_ via the synergy of adsorption and photocatalysis under visible light. Chem. Eng. J..

[56] Du C., Xu J., Ding G., He D., Zhang H., Qiu W., Li C., Liao G. (2023). Recent Advances in LDH/g-C_3_N_4_ Heterojunction Photocatalysts for Organic Pollutant Removal. Nanomaterials.

[57] Zhu B., Xia P., Ho W., Yu J. (2015). Isoelectric point and adsorption activity of porous g-C_3_N_4_. Appl. Surf. Sci..

[58] Yang Y.J., Li W. (2018). Ultrasonic assisted coating of multiwalled carbon nanotubes with NiFe-layered double hydroxide for improved electrocatalytic oxygen reduction. J. Electroanal. Chem..

[59] Khan A.A., Tahir M. (2025). Self-assembled 2D/2D Z-Scheme heterojunction of NiAl-LDH/protonated g-C_3_N_4_ on conductive 2D V2C MXene for high-performance solar-driven photocatalytic CO_2_ to fuel conversion. Fuel.

[60] Zhou A.Q., Yang J.M., Zhu X.W., Zhu X.L., Liu J.Y., Zhong K., Chen H.X., Chu J.Y., Du Y.S., Song Y.H. (2022). Self-assembly construction of NiCo LDH/ultrathin g-C_3_N_4_ nanosheets photocatalyst for enhanced CO_2_ reduction and charge separation mechanism study. Rare Met..

[61] Khan A., Ansari M.A., Onaizi S.A. (2025). Photocatalytic and Electrocatalytic Conversion of CO_2_ into Valuable Chemicals Using Layered Double Hydroxide-Based Materials: A Review. Surf. Interfaces.

[62] Arif M., Yasin G., Shakeel M., Fang X., Gao R., Ji S., Yan D. (2018). Coupling of bifunctional CoMn-layered double hydroxide@ graphitic C_3_N_4_ nanohybrids towards efficient photoelectrochemical overall water splitting. Chem. Asian J..

[63] Yuan X., Li W. (2017). Graphitic-C_3_N_4_ modified ZnAl-layered double hydroxides for enhanced photocatalytic removal of organic dye. Appl. Clay Sci..

[64] Liu J., Li J., Bing X., Ng D.H., Cui X., Ji F., Kionga D.D. (2018). ZnCr-LDH/N-doped graphitic carbon-incorporated g-C_3_N_4_ 2D/2D nanosheet heterojunction with enhanced charge transfer for photocatalysis. Mater. Res. Bull..

[65] Sahoo D.P., Das K.K., Patnaik S., Parida K. (2020). Double charge carrier mechanism through 2D/2D interface-assisted ultrafast water reduction and antibiotic degradation over architectural S, P co-doped g-C_3_N_4_/ZnCr LDH photocatalyst. Inorg. Chem. Front..

[66] Chowdhury S., Nugraha A.S., Yuliarto B., Yamauchi Y., Kaneti Y.V. (2025). Nanoarchitecturing of Bimetallic Metal—Organic Frameworks for Emerging Applications in Quartz Crystal Microbalance Gas Sensors. Small Methods.

[67] Arif M., Yasin G., Shakeel M., Mushtaq M.A., Ye W., Fang X., Ji S., Yan D. (2019). Hierarchical CoFe-layered double hydroxide and g-C_3_N_4_ heterostructures with enhanced bifunctional photo/electrocatalytic activity towards overall water splitting. Mater. Chem. Front..

[68] Mruthunjayappa M.H., Kotrappanavar N.S., Mondal D. (2022). New prospects on solvothermal carbonisation assisted by organic solvents, ionic liquids and eutectic mixtures–A critical review. Prog. Mater. Sci..

[69] Gu Q., Feng C., Liu C., Rong J., Zhang Y., Zheng X., Li Z., Xu S. (2024). Constructing direct Z-Scheme heterojunction of NiCo-LDH coated with g-C_3_N_4_ for boosting photocatalytic H_2_ evolution. Fuel.

[70] Srisuvetha V.T., Prakash S., Vallalperuman K. (2025). Synthesis and hydrogen production performance of g-C_3_N_4_/CoAl-LDH heterojunction photocatalyst by facile hydrothermal route. J. Mater. Sci. Mater. Electron..

[71] Sheikhpoor H., Saljooqi A., Shamspur T., Mostafavi A. (2021). Co-Al Layered double hydroxides decorated with CoFe_2_O_4_ nanoparticles and g-C3N4 nanosheets for efficient photocatalytic pesticide degradation. Environ. Technol. Innov..

[72] Zhang L., Wang Z., Fang P., Wang W., Sun W. (2021). Hierarchical Sheet-on-Sheet ZnTi-LDHs/g-C_3_N_4_ composites with enhanced photocatalytic activity prepared by mechanical mixing. Appl. Clay Sci..

[73] Sherryna A., Tahir M., Zakaria Z.Y. (2024). Trimetallic Ni_x_Co_y_Al_z_ LDH-Modified g-C_3_N_4_ with Influential Effects of M^2+^/M^3+^ (Ni/Co) Active Centers for Stimulating Photocatalytic Hydrogen Production. ACS Appl. Energy Mater..

[74] Alaghmandfard A., Ghandi K. (2022). A Comprehensive Review of Graphitic Carbon Nitride (g-C_3_N_4_)–Metal Oxide-Based Nanocomposites: Potential for Photocatalysis and Sensing. Nanomaterials.

[75] Paul D.R., Sharma R., Nehra S.P., Sharma A. (2019). Effect of calcination temperature, pH and catalyst loading on photodegradation efficiency of urea derived graphitic carbon nitride towards methylene blue dye solution. RSC Adv..

[76] Zhou Y., Wu Y., Wu H., Xue J., Ding L., Wang R., Wang H. (2022). Fast hydrogen purification through graphitic carbon nitride nanosheet membranes. Nat. Commun..

[77] Wang P., Ng D.H.L., Zhou M., Li J. (2019). Freely standing MgAl-layered double hydroxides nanosheets and their derived metal oxides on g-C_3_N_4_ thin-layer designed for obtaining synergic effect of adsorption and photocatalysis. Appl. Clay Sci..

[78] Li X., Xue J., Ma S., Xu P., Huang C., Wang M. (2019). Synthesis of MgAl LDH/Acidified g-C_3_N_4_ heterojunction photocatalyst for improved tetracycline hydrochloride degradation activity. Nano.

[79] Gandamalla A., Manchala S., Verma A., Fu Y.P., Shanker V. (2021). Microwave-assisted synthesis of ZnAl-LDH/g-C_3_N_4_ composite for degradation of antibiotic ciprofloxacin under visible-light illumination. Chemosphere.

[80] Maridevaru M.C., Al Marzouqi F., Nagaveni M., Kumari M.M., Shankar M.V., Khraisheh M., Selvaraj R. (2025). Eu_2_O_3_/CoMnAl-LDH@ g-C_3_N_4_ ternary S-Scheme heterojunction photocatalyst for hydrogen production under solar light illumination. Int. J. Hydrogen Energy.

[81] Li X., Yu Z., Shao L., Zeng H., Liu Y., Feng X. (2020). A novel strategy to construct a visible-light-driven Z-Scheme (ZnAl-LDH with active phase/g-C_3_N_4_) heterojunction catalyst via polydopamine bridge (a similar “bridge” structure). J. Hazard. Mater..

[82] Alam K., Khan K.I., Mehdi M.S., Wahab A., Haider S., Stoller M. (2025). Advanced Synthesis of Ag-Doped g-C_3_N_4_/NiFe-LDH Photocatalyst via Spin Disc Reactor for Enhanced RhB Dye Degradation. Catal. Lett..

[83] Maridevaru M.C., Al Marzouqi F., Nagaveni M., Kumari M.M., Shankar M.V., Selvaraj R. (2025). Sunlight driven Eu_2_O_3_/CoNiZn-LDH@ g-C_3_N_4_ ternary heterojunction nanocomposite photocatalyst for hydrogen generation. Renew. Energy.

[84] Yan J., Zhang X., Zheng W., Lee L.Y.S. (2021). Interface engineering of a 2D-C_3_N_4_/NiFe-LDH heterostructure for highly efficient photocatalytic hydrogen evolution. ACS Appl. Mater. Interfaces.

[85] Tonda S., Kumar S., Bhardwaj M., Yadav P., Ogale S. (2018). g-C_3_N_4_/NiAl-LDH 2D/2D hybrid heterojunction for high-performance photocatalytic reduction of CO_2_ into renewable fuels. ACS Appl. Mater. Interfaces.

[86] Huang M., Yang Z., Lu L., Xu J., Wang W., Yang C. (2022). The preparation of g-C_3_N_4_/CoAl-LDH nanocomposites and their depollution performances in cement mortars under UV-visible light. Catalysts.

[87] Zhang H., Li M., Li N., Jiang R., Yin E., Li X. (2025). Performance enhancement and mechanism of tetracycline removal by visible light-driven photo bio-electro-fenton system with CoFe-LDH/g-C_3_N_4_ cathode. J. Environ. Manag..

[88] Yang Y., Wu J., Xiao T., Tang Z., Shen J., Li H., Zhou Y., Zou Z. (2019). Urchin-like hierarchical CoZnAl-LDH/RGO/g-C_3_N_4_ hybrid as a Z-Scheme photocatalyst for efficient and selective CO_2_ reduction. Appl. Catal. B Environ..

[89] Arjomandi-Behzad L., Alinejad Z., Zandragh M.R., Golmohamadi A., Vojoudi H. (2023). Facile synthesis of hollow spherical g-C_3_N_4_@ LDH/NCQDs ternary nanostructure for multifunctional antibacterial and photodegradation activities. iScience.

[90] Luo J., Liu Y., Fan C., Tang L., Yang S., Liu M., Wang M., Feng C., Ouyang X., Wang L. (2021). Direct attack and indirect transfer mechanisms dominated by reactive oxygen species for photocatalytic H_2_O_2_ production on g-C_3_N_4_ possessing nitrogen vacancies. ACS Catal..

[91] Usman M., Khan K.I., Khalid S., Saeed F., Wahab A., Asghar M.S., Althobaiti A., Mohammad A., Dai H. (2025). Synergistic defect engineering and *Z*-Scheme heterojunction in ultrasonication assisted CuFe LDH and carbon vacancies modified g-C_3_N_4_ composite for electrocatalytic water splitting and photocatalytic dye degradation. J. Water Process Eng..

[92] Zheng J., Fan C., Li X., Yang Q., Wang D., Duan A., Pan S., Zhang B., Ding J., Rong S. (2023). Effective mineralization and detoxification of tetracycline hydrochloride enabled by oxygen vacancies in g-C_3_N_4_/LDH composites. Sep. Purif. Technol..

[93] Yang D., Wang Y., Zhao J., Dai J., Yan Y., Chen L., Ye J. (2023). Strong coupling of super-hydrophilic and vacancy-rich g-C_3_N_4_ and LDH heterostructure for wastewater purification: Adsorption-driven oxidation. J. Colloid Interface Sci..

[94] Mutahir S., Khan M.A., Noor W., Butt R., Elkholi S.M., Bououdina M., Alsuhaibani A.M., Munawar K.S., Humayun M. (2024). Oxygen doped g-C_3_N_4_/LDH composite as highly efficient photocatalyst for wastewater treatment. Z. Für Phys. Chem..

[95] Zheng W., Ye C., Yu M., Yang S., Xiu Y., He X., Xue H., Xia J., Gao R., Yuan Z. (2025). Fabrication of metal-doped graphite phase carbon nitride-based membrane and its application. Adv. Compos. Hybrid Mater..

[96] Starukh H., Praus P. (2020). Doping of graphitic carbon nitride with non-metal elements and its applications in photocatalysis. Catalysts.

[97] Bilal A., Bahadur A., Khan M.S., Khan I., Ali S., Iqbal S., Asimov A., Alhabradi M., Alruwaili M., Mahmood S. (2025). A high-performance S-Scheme based Ti_3_C_2_T_x_ Mxene-enhanced Sg-C_3_N_4_/NiAl layered double hydroxide ternary heterojunction photocatalyst for sustainable hydrogen production. J. Power Sources.

[98] Hu J., Sun C., Wu L., Zhao G., Liu H., Jiao F. (2023). Halogen doped g-C_3_N_4_/ZnAl-LDH hybrid as a Z-Scheme photocatalyst for efficient degradation for tetracycline in seawater. Sep. Purif. Technol..

[99] Usman M., Imran Khan K., Adnan M., Khan A. (2024). Facile synthesis of NiAl-LDH/Ag/g-C_3_N_4_ ternary composite for photocatalytic degradation of methylene blue. Fuller. Nanotub. Carbon Nanostruct..

[100] Kaur J., Pal B., Singh S., Kaur H. (2023). Fabrication of highly efficient Au and layered double hydroxide modified g-C_3_N_4_ ternary composites for degradation of pharmaceutical drug: Pathways and mechanism. Surf. Interfaces.

[101] Niknam M., Vandchali M.B., Ghasemi E., Kazemi A., Yousefi-Limaee N. (2025). Synthesis and characterization of a novel g-C_3_N_4_/NiAl-LDH/CeO_2_ photocatalyst for degradation of rhodamine B. Int. J. Environ. Sci. Technol..

[102] Jiang H., He J., Deng C., Hong X., Liang B. (2022). Advances in Bi_2_WO_6_-based photocatalysts for degradation of organic pollutants. Molecules.

[103] Zhang M., Duo F., Lan J., Zhou J., Chu L., Wang C., Li L. (2023). In situ synthesis of a Bi_2_O_3_ quantum dot decorated BiOCl heterojunction with superior photocatalytic capability for organic dye and antibiotic removal. RSC Adv..

[104] Lin H., Xiao Y., Geng A., Bi H., Xu X., Xu X., Zhu J. (2022). Research progress on graphitic carbon nitride/metal oxide composites: Synthesis and photocatalytic applications. Int. J. Mol. Sci..

[105] Estrada-Movilla E., Castillo-Saenz J., Valdez-Salas B., Ortiz-Pérez Á., Beltrán-Partida E., Salvador-Carlos J., Puello-Polo E. (2025). Challenges and Opportunities for g-C_3_N_4_-Based Heterostructures in the Photodegradation of Environmental Pollutants. Catalysts.

[106] Meng F., Qin Y., Lu J., Lin X., Meng M., Sun G., Yan Y. (2021). Biomimetic design and synthesis of visible-light-driven g-C_3_N_4_ nanotube@ polydopamine/NiCo-layered double hydroxides composite photocatalysts for improved photocatalytic hydrogen evolution activity. J. Colloid Interface Sci..

[107] Hasija V., Nguyen V.H., Kumar A., Raizada P., Krishnan V., Khan A.A.P., Singh P., Lichtfouse E., Wang C., Huong P.T. (2021). Advanced activation of persulfate by polymeric g-C_3_N_4_ based photocatalysts for environmental remediation: A review. J. Hazard. Mater..

[108] Maridevaru M.C., Nagaveni M., Kumari M.M., Shankar M.V., Khraisheh M., Selvaraj R. (2024). Unravelling the efficiency of CoAl-LDH/g-C_3_N_4_ nanosheets: Type-II heterojunction for photocatalytic hydrogen production and dye degradation under solar light irradiation. Int. J. Hydrogen Energy.

[109] Nadikatla S.K., Chintada V.B., Gurugubelli T.R., Koutavarapu R. (2023). Review of recent developments in the fabrication of ZnO/CdS heterostructure photocatalysts for degradation of organic pollutants and hydrogen production. Molecules.

[110] Ran J., Talebian-Kiakalaieh A., Zhang S., Hashem E.M., Guo M., Qiao S.Z. (2024). Recent advancement on photocatalytic plastic upcycling. Chem. Sci..

[111] Li S., Xu W., Meng L., Tian W., Li L. (2021). Recent Progress on Semiconductor Heterojunction-Based Photoanodes for Photoelectrochemical Water Splitting, Small. Science.

[112] Low J., Yu J., Jaroniec M., Wageh S., Al-Ghamdi A.A. (2017). Heterojunction photocatalysts. Adv. Mater..

[113] Sun H., Tillotson M.R., Wang D., Zhao X. (2024). A novel Z-Scheme SnS/NiAl-LDH/g-C_3_N_4_ heterojunction for piezo-photocatalytic degradation of tetracycline: Performance and mechanism. Surf. Interfaces.

[114] Hu J., Zhao G., Wu L., Sun C., Long X., Long X., Jiao F. (2022). Designing and fabricating a vulcanized ZnAl LDH-modified g-C_3_N_4_ heterojunction for enhanced visible-light-driven photocatalytic degradation activity. Ind. Eng. Chem. Res..

[115] Nong J., Jin Y., Tan J., Ma H., Lian Y. (2022). Construction of NiCo-LDH/g-C_3_N_4_ heterojunctions as efficient photocatalysts for enhanced degradation of tetracycline hydrochloride and hydrogen evolution. New J. Chem..

[116] Liu H., Zhu S., Zhi Y., Yue H., Liu X. (2025). Donor-acceptor type covalent organic frameworks: Design, optimization strategies, and applications. Chem. Sci..

[117] Sun X., Li L., Hu T., Lian Q., Liu Y., Hou J., Yu Y., Cao Y., Ma F. (2023). In_2_S_3_/g-C_3_N_4_/CoZnAl-LDH composites with the lamellar dual S-Scheme heterostructure and its enhanced photocatalytic performance. Colloids Surf. A Physicochem. Eng. Asp..

[118] Huang Y., Yu J., Wu Z., Li B., Li M. (2024). All-inorganic lead halide perovskites for photocatalysis: A review. RSC Adv..

[119] Feng X., Li X., Su B., Ma J. (2022). Hydrothermal construction of flower-like g-C_3_N_4_/NiZnAl-LDH S-Scheme heterojunction with oxygen vacancies for enhanced visible-light triggered photocatalytic performance. J. Alloys Compd..

[120] Wu X., Tan L., Chen G., Kang J., Wang G. (2024). G-C_3_N_4_-based S-Scheme heterojunction photocatalysts. Sci. China Mater..

[121] Thirugnanam B., Mani P., Settu M., Venkattappan A. (2025). Unlocking the potential of photocatalysts: Recent advances in layered double hydroxide and future outlook. J. Environ. Sci..

[122] Jie Z., Yang L., Huiyuan T., Mengyan X., Xiuhong D., Zehua W., Chunguang L., Xianying D., Jiehu C. (2023). Layered by layered construction of three novel ZnCo-LDHs/g-C_3_N_4_ for the removal of sunset yellow by adsorption-photocatalytic process. Environ. Sci. Pollut. Res..

[123] Weidner E., Karbassiyazdi E., Altaee A., Jesionowski T., Ciesielczyk F. (2022). Hybrid metal oxide/biochar materials for wastewater treatment technology: A review. ACS Omega.

[124] Anandraj F.N., Panda T.K., Thangarasu S., Palanisamy G., Neerugatti K.E. (2025). Persulfate salts to combat bacterial resistance in the environment through antibiotic degradation and biofilm disruption. Water Res..

[125] Gao J., Zhao X., Li C., Luo Z., Zhang L., Xiao Z., Mu T., Liu F., Gao R., Zhang J. (2025). Efficient atrazine degradation through ZnFe_2_O_4_-catalyzed peroxymonosulphate activation. Sci. Rep..

[126] Soltani R.D.C., Abolhasani E., Mashayekhi M., Jorfi N., Boczkaj G., Khataee A. (2022). Degradation of tetracycline antibiotic utilizing light driven-activated oxone in the presence of g-C_3_N_4_/ZnFe LDH binary heterojunction nanocomposite. Chemosphere.

[127] Yin C., Liu Y., Hu T., Chen X. (2025). Graphitic Carbon Nitride Nanomaterials-Based Electrochemical Sensing Interfaces for Monitoring Heavy Metal Ions in Aqueous Environments. Nanomaterials.

[128] Nhung N.T.H., Rabani I., Tran N.T., Thuy B.T.P., Truong H.B. (2025). Advances in Co_3_O_4_ nanomaterial-based photocatalysts for water purification: Mechanisms, green synthesis, activation of oxidants, waste-derived sources, and computational insights. RSC Adv..

[129] Zhong X., Liu X., Ji M., Jiang F. (2025). Densely Stacked CoCu-MOFs Coated with CuAl/LDH Enhance Sulfamethoxazole Degradation in PMS-Activated Systems. Nanomaterials.

[130] Wu P., Qin Y., Gao M., Zheng R., Zhang Y., Li X., Liu Z., Zhang Y., Cao Z., Liu Q. (2024). Broad Spectral Response FeOOH/BiO_2−x_ Photocatalyst with Efficient Charge Transfer for Enhanced Photo-Fenton Synergistic Catalytic Activity. Molecules.

[131] Zeng H., Zhang H., Deng L., Shi Z. (2020). Peroxymonosulfate-assisted photocatalytic degradation of sulfadiazine using self-assembled multi-layered CoAl-LDH/g-C_3_N_4_ heterostructures: Performance, mechanism and eco-toxicity evaluation. J. Water Process Eng..

[132] Shen Y., de Vidales M.J.M., Gorni G., Gomez-Herrero A., Fernandez-Martinez F., Dos santos-Garcia A.J. (2022). Enhanced performance and recyclability for peroxymonosulfate activation via g-C_3_N_4_ supported CoFe layer double oxide. Chem. Eng. J..

[133] Wei Z., Ren X., Mei T., Xiang R., Wei W., Yang X., Fang Y., Xu W., Zhu J., Liang J. (2025). MgFeTi-LDH/g-C_3_N_4_ composites promote sodium persulfate activation through Fe^3+^/Fe^2+^ cycles for efficient tetracycline degradation under visible light irradiation. New J. Chem..

[134] Qin Q., Xu H., Sun S., Zhao Z., Ren X., Li M., Song B., Shao G., Wang H., Lu H. (2025). Facile synthesis of MgAl-LDH/g-C_3_N_4_ composites for the photocatalytic degradation toward ciprofloxacin. J. Environ. Chem. Eng..

[135] Tripathi A., Narayanan S. (2020). Stimulation of n-π* transition of g-C_3_N_4_ through ZnAl-layered double hydroxide for solar light assisted phenol degradation. Mater. Sci. Semicond. Process..

[136] Lestari P.R., Takei T., Kumada N. (2021). Novel ZnTi/C_3_N_4_/Ag LDH heterojunction composite for efficient photocatalytic phenol degradation. J. Solid State Chem..

[137] Rath A., Sahu P.K., Champati A., Pradhan A., Madhual A., Mishra P.M., Naik B. (2025). A novel Cu-Al LDH/g-C_3_N_4_ Z-Scheme photocatalyst for environmental remediation of cresol red. Discov. Appl. Sci..

[138] Huang X., Xu X., Yang R., Fu X. (2022). Synergetic adsorption and photocatalysis performance of g-C_3_N_4_/Ce-doped MgAl-LDH in degradation of organic dye under LED visible light. Colloids Surf. A Physicochem. Eng. Asp..

[139] Liu Q., Deng W., Zhang H., Fang J., Xie Y., Liu C., Han X., Xu X., Zhou Z. (2025). Enhanced Photocatalytic Activity of CQDs-Modified Layered g-C_3_N_4_/Flower-like ZnO Heterojunction for Efficient Degradation of Ciprofloxacin. Nanomaterials.

[140] Elmetwalli A., Allam N.G., Hassan M.G., Albalawi A.N., Shalaby A., El-Said K.S., Salama A.F. (2023). Evaluation of Bacillus aryabhattai B_8_W_22_ peroxidase for phenol removal in waste water effluents. BMC Microbiol..

[141] Zhang L., Tang W., Ma T., Zhou L., Hui C., Wang X., Wang P., Zhang C., Chen C. (2019). Laccase-immobilized tannic acid-mediated surface modification of halloysite nanotubes for efficient bisphenol-A degradation. RSC Adv..

[142] Li C., Wang Y., Zhao G., Yan T., Zhang T., Liu L., Jiao F., Huang J. (2020). A new porous Ag_3_PO_4_/(Cs, Rb)_x_WO_3_/g-C_3_N_4_/CoAl-LDH composite towards efficient photocatalytic degradation of phenol and its derivatives. Desalination Water Treat..

[143] Rosa D., Remmani R., Bavasso I., Bracciale M.P., Di Palma L. (2025). Biochar supported Fe-TiO_2_ composite for wastewater treatment: Solid-state synthesis and mechanistic insights. Chem. Eng. Sci..

